# Association Between Switching to a High-Deductible Health Plan and Major Cardiovascular Outcomes

**DOI:** 10.1001/jamanetworkopen.2020.8939

**Published:** 2020-07-24

**Authors:** J. Frank Wharam, Jamie Wallace, Fang Zhang, Xin Xu, Christine Y. Lu, Adrian Hernandez, Dennis Ross-Degnan, Joseph P. Newhouse

**Affiliations:** 1 Division of Health Policy and Insurance Research, Department of Population Medicine, Harvard Medical School and Harvard Pilgrim Health Care Institute, Boston, Massachusetts; 2 Department of Medicine, Duke University School of Medicine, Durham, North Carolina; 3 Department of Health Care Policy, Harvard Medical School, Boston, Massachusetts; 4 Department of Health Policy and Management, Harvard T.H. Chan School of Public Health, Boston, Massachusetts; 5 Harvard Kennedy School, Cambridge, Massachusetts; 6 National Bureau of Economic Research, Cambridge, Massachusetts

## Abstract

**Importance:**

Most people with commercial health insurance in the US have high-deductible plans, but the association of such plans with major health outcomes is unknown.

**Objective:**

To describe the association between enrollment in high-deductible health plans and the risk of major adverse cardiovascular outcomes.

**Design, Setting, and Participants:**

This cohort study examined matched groups before and after an insurance design change. Data were from a large national commercial (and Medicare Advantage) health insurance claims data set that included members enrolled between January 1, 2003, and December 31, 2014. The study group included 156 962 individuals with risk factors for cardiovascular disease who were continuously enrolled in low-deductible (≤$500) health plans during a baseline year followed by up to 4 years in high-deductible (≥$1000) plans with typical value-based features after an employer-mandated switch. The matched control group included 1 467 758 individuals with the same risk factors who were contemporaneously enrolled in low-deductible plans. Data were analyzed from December 2017 to March 2020.

**Exposures:**

Employer-mandated transition to a high-deductible health plan.

**Main Outcomes and Measures:**

Time to first major adverse cardiovascular event defined as myocardial infarction or stroke.

**Results:**

The study group included 156 962 individuals and the control group included 1 467 758 individuals; the mean age of members was 53 years (SD: high-deductible group, 6.7 years; control group, 6.9 years), 47% were female, and approximately 48% lived in low-income neighborhoods. First major adverse cardiovascular events among high-deductible health plan members did not differ relative to controls at follow-up vs baseline (adjusted hazard ratio, 1.00; 95% CI, 0.89-1.13). Findings were similar among subgroups with diabetes (adjusted hazard ratio, 0.93; 95% CI, 0.75-1.16) and with other cardiovascular risk factors (adjusted hazard ratio, 0.93; 95% CI, 0.81-1.07).

**Conclusions and Relevance:**

Mandated enrollment in high-deductible health plans with typical value-based features was not associated with increased risk of major adverse cardiovascular events.

## Key Points

**Question:**

Are high-deductible health plans associated with an increased risk of major cardiovascular events?

**Finding:**

This cohort study included 156 962 individuals with cardiovascular disease risk factors who experienced mandated enrollment in health insurance plans with high deductibles but relatively low medication costs, a common value-based feature. Members with high-deductible health plans did not have detectable increases in major adverse cardiovascular events compared with 1 467 758 members with low-deductible health plans.

**Meaning:**

Among patients with cardiovascular disease risk factors in this study, enrollment in typical high-deductible health plans was not associated with increased risk of major adverse cardiovascular events during 4 follow-up years.

## Teaser

This cohort study assesses whether an employer-mandated switch from a low-deductible health plan to high-deductible health plan is associated with the risk of major adverse cardiovascular outcomes among more than 1.5 million individuals in the US.

## Introduction

Cardiovascular disease kills more people in the US than any other condition, accounting for 30% of deaths in 2017.^1^ Decades of improvements in cardiovascular mortality began slowing in about 2010,^2,3,4,5,6^ and major adverse cardiovascular events, such as stroke and myocardial infarction, began increasing among adults under age 65.^7^ The reasons for these trends are unclear, but experts have proposed causes such as the earlier onset of cardiovascular risk factors,^2^ stagnation of preventive care,^7^ and less generous health insurance coverage in the US.^7^

Concerns about the contribution of health insurance to these trends are based on the rapid expansion of high-deductible health plans (HDHPs) and previous research about the health effects associated with high out-of-pocket costs.^8,9,10,11^ High-deductible plans, which require potential annual out-of-pocket spending of approximately $1000 to $7000 per person for most nonpreventive care, now cover most commercially insured people in the US. In 2018, 58% of workers with individual plans had deductibles of $1000 or higher and 26% had deductibles of $2000 or higher.^12^ However, most employer-sponsored HDHPs include several value-based insurance design features,^13^ such as no or relatively low cost sharing for medications, secondary preventive testing, and annual physical examination visits.^12^

Although no research has examined the association between high out-of-pocket costs and adverse cardiovascular events, several previous studies have raised concerns. The RAND Health Insurance Experiment of the 1970s to 1980s detected suboptimal blood pressure control among the poorest and sickest individuals subject to high cost sharing, and investigators predicted a 16% mortality increase.^8^ More recent research detected adverse short-term health outcomes among low-income patients with diabetes in HDHPs^9,10^ as well as delays in receiving major cardiovascular care.^11^ We hypothesized that people with risk factors for cardiovascular disease would experience increases in major adverse cardiovascular events after an employer-mandated switch from low-deductible health plans to HDHPs relative to people who remained in low-deductible plans.

## Methods

### Study Population

We drew our study population from approximately 48 million commercially insured members in a large national commercial (and Medicare Advantage) health insurance claims data set of members enrolled between January 1, 2003, and December 31, 2014. The data set contains enrollment information and all medical, pharmacy, and hospitalization claims. We included only members with employer-sponsored insurance. This study followed the Strengthening the Reporting of Observational Studies in Epidemiology (STROBE) reporting guideline for cohort studies. This study was approved by the institutional review board of Harvard Pilgrim Health Care Institute with a waiver for the requirement of obtaining patient informed consent because the data are deidentified and the research would not be feasible otherwise.

We considered an insurance plan to have a low deductible if the annual level was $500 or lower and to be a HDHP if the annual amount was $1000 or higher. For employers with fewer employees, we determined the deductible amount from a benefits table obtained from the health insurer. This table mostly included employers with fewer than 100 individuals but also had a modest number of larger employers. For employers not represented in the insurance benefits table (mostly employers with a large number of employees), we imputed deductible amounts from actual out-of-pocket spending by individuals who used health services, applying an algorithm with sensitivity and specificity rates higher than 95% (eTable 1 in the Supplement).^11^

Individuals in our study were unable to choose between low-deductible health plans and HDHPs because we included employers who offered only 1 deductible level each year. Some employers offered a low-deductible plan for the duration of the study, and others offered only a low-deductible plan and then switched all enrollees to a HDHP.

We defined the index date for employers that switched to HDHPs as the beginning of the month when the switch occurred. For employers that did not switch plans, the index date was the beginning of the month when their yearly account renewed. Some members had multiple eligible index dates (eg, multiple low-to-low deductible years or both low-to-low and low-to-HDHP years). In the cases of members with both low-to-low and low-to-HDHP years, we randomly assigned enrollees to the HDHP pool or the control pool. For members assigned to the control pool that had multiple low-to-low deductible spans, we randomly selected one of their potential index dates (and their corresponding before-after enrollment years). Employers had index dates between January 1, 2004, and December 1, 2014.

For all individuals in the study, the beginning of the study period (time zero) was 12 months before the employer’s index date, and we defined this 12-month period as the baseline year (eFigure 1 in the Supplement). The employer’s index date was the beginning of the follow-up period. For each individual, we measured months from time zero to the first outcome in the baseline period, and we measured months from the index date to the first outcome in the follow-up period.

Individuals were eligible for the study based on the following criteria: enrolled through an employer that had coverage for at least 1 year before and after the index date and were aged 40 to 64 years; met criteria for having diabetes, cardiovascular disease, hypertension, or hyperlipidemia before the index date (based on version 11.1 of the Johns Hopkins ACG System)^14,15^; and continuously enrolled for at least 1 year before and at least 1 month after the index date (eFigure 1 in the Supplement).

We also created 4 subgroups for sensitivity analyses. To determine whether patients with certain comorbidities were at higher risk of adverse cardiovascular events under HDHPs, we identified 2 mutually exclusive subgroups (1) with diabetes (eTable 2 in the Supplement) and (2) with other cardiovascular risk factors (established cardiovascular disease, hypertension, or hyperlipidemia; eTable 3 in the Supplement). We also identified a very high-risk subgroup (3) with diabetes, established cardiovascular disease, and either hyperlipidemia or hypertension. To determine whether effect estimates varied over time, we also identified a group (4) with index dates in 2009 and after.

Most HDHP group members (approximately 90% per follow-up year) did not have health savings account–eligible plans and therefore had medications subject to traditional tiered copayments rather than to the deductible.^16^ Control group members had a tiered copayment structure for medications.

### Study Design

We compared matched cohorts in this observational, before-after study. All members were enrolled in a commercial health insurance plan between January 1, 2003, and December 31, 2014. The intervention group consisted of individuals in low-deductible health insurance plans for 1 year who were switched to HDHPs and were then enrolled for an additional 1 month to 4 years (eFigure 1 in the Supplement). The control group included contemporaneously enrolled matched individuals who remained in low-deductible health plans throughout the study. We matched based on the year of the index date; the propensity of the employer to mandate HDHPs and the propensity of individuals to work for such employers (each divided into tertiles)^17,18^ (eAppendix in the Supplement); the presence of diabetes, cardiovascular disease, hypertension, or hyperlipidemia during or before the baseline year (yes or no); baseline occurrence of stroke, myocardial infarction, or amputation (yes or no); and follow-up duration category (years of follow-up).

We used coarsened exact matching (eAppendix in the Supplement),^19,20,21^ an approach that is similar to exact matching but differs by using categories instead of exact values to match. The software for coarsened exact matching creates weights for each stratum that adjust for any differences between study groups in the proportion of individuals in the stratum. We ran separate matches for the overall cohort and for the 4 key subgroups already described.

### Outcome Measures

Our primary outcome was time to first major adverse cardiovascular event, a composite measure of myocardial infarction or stroke. For secondary measures, we analyzed time to first myocardial infarction, stroke, and amputation separately. We defined myocardial infarction as a hospitalization of 3 to 180 days that had *International Classification of Diseases, Ninth Revision, Clinical Modification* relevant discharge diagnoses (eTable 4 in the Supplement) in the first or second position.^22^ We used a similar hospital-based stroke algorithm (eTable 4 in the Supplement).^23^ We defined extremity amputation based on procedure codes listed in eTable 4 in the Supplement.

In sensitivity analyses, we added all-cause mortality (from the Social Security Administration’s Death Master file)^24^ to our composite major adverse cardiovascular events measure in the follow-up period because HDHPs could accelerate time to death. We assessed this measure in the entire cohort as well as among a cohort with follow-up time censored after November 2011 given that the Death Master file became incomplete after this time.^25^

Finally, we conducted a post hoc analysis of prescription fills to determine whether stable rates of cardioprotective medication use among HDHP members might be associated with minimal changes in adverse cardiovascular events. We assessed number of 30-day equivalent prescription fills per year of antihypertensives and lipid-lowering medications. Among patients with diabetes, we also examined use of oral antidiabetes medications and insulin.

### Covariates

We applied the Johns Hopkins ACG System^14,15^ in calculating members’ baseline period morbidity score (eAppendix in the Supplement). We used American Community Survey data^26^ to characterize census tracts^27^ in which members resided. Categories included neighborhoods with below-poverty levels of lower than 5%, 5% to 9.9%, 10% to 19.9%, and 20% or higher and with educational levels below high school of lower than 15%, 15% to 24.9%, 25% to 39.9%, and 40% or higher.^28^ We used geocoding to classify participants as from white, black, Hispanic, or mixed race/ethnicity neighborhoods, and we used a superseding Hispanic or Asian race/ethnicity categorization based on the E-Tech system (Ethnic Technologies) that analyzes full names and geographic locations of individuals.^29^ Other covariates included age category (40-49, 50-59, and 60-64 years); sex; US region (West, Midwest, South, and Northeast); employer size used as either a continuous variable or with categories of 0 to 99, 100 to 999, or 1000 or more individuals (eAppendix in the Supplement); and calendar month of the index date.

### Statistical Analysis

We compared baseline characteristics of our study groups using standardized differences.^30^ We analyzed time to the primary and disaggregated secondary outcomes in Cox proportional hazards regression models adjusted for sex, employer size, race/ethnicity, educational level, poverty, and US region. We censored individuals if they dropped from the sample (eg, as a result of disenrollment), reached age 65 years (when Medicare coverage begins), or reached the end of the baseline or follow-up period (ie, 4 years after the index date).

We analyzed time to event in the baseline and the follow-up periods in a single model (eAppendix in the Supplement), and the key term of interest was an interaction between study period (baseline or follow-up) and study group (HDHP or control). This term generated a HDHP:control hazard ratio at follow-up adjusted for the HDHP:control hazard ratio at baseline and also adjusted for the other terms in the model. Our primary effect estimate was thus an adjusted hazard ratio of ratios, which we term *adjusted hazard ratio* (aHR) given that the baseline hazard ratio can be considered another variable that adjusts the follow-up period hazard ratio.

We used the same analytic approach in the 4 subgroups of interest. Our approach was also similar in the overall group after adding all-cause mortality to the composite outcome, but we used a post-only model with *study group* as the term of interest. That is, we assessed this measure only at follow-up because our study design required patients to be alive for the entire baseline.

In post hoc analyses of cardioprotective medication use, we used a generalized estimating equations regression model with a zero-inflated negative binomial distribution, adjusting for sex, employer size, race/ethnicity, educational level, poverty, and US region. The term of interest was an interaction between study year (years 0-4 as separate categories) and study group, and we used marginal effects methods^31^ to calculate relative and absolute changes in the HDHP group compared with the control group from baseline to follow up. All analyses were conducted from December 2017 to March 2020 with SAS Studio, version 3.7 (SAS Institute Inc) or STATA, version 15 (StataCorp).

## Results

The matched cohort included 156 962 HDHP members and 1 467 758 control members. The HDHP group sample sizes were 30 449 with diabetes (eTable 2 in the Supplement), 127 667 with other cardiovascular risk factors (eTable 3 in the Supplement), and 4085 in the very high-risk subgroup. The intervention and control group members’ mean age was 53 years (SD: high-deductible group, 6.7 years; control group, 6.9 years), 47% were female, 48% to 49% lived in neighborhoods with below-poverty levels of 10% or higher, 9% to 9% lived in neighborhoods in which 25% or higher of the individuals had educational levels below high school, and 8% were Hispanic. For both groups, the percentage with a follow-up time of 1 year was 78.0%; 2 years, 46.5%; 3 years, 29.1%; and 4 years, 18.8%. Most dropout was related to member or employer disenrollment, or employers adopting a deductible level that made enrollees ineligible to be in their respective study group. Less than half a percent in each group experienced dropout due to death. After matching and applying match-generated weights, all standardized differences between the intervention group and the control group at baseline were lower than 0.2 (Table), indicating minimal differences.^30^

**Table.** Baseline Characteristics of the Study Groups Before and After Matching

Characteristic	Unmatched, No. (%)		Standardized difference^a^	Matched, No. (%)		Standardized difference^a^
	HDHP group (n = 158 179)	Control group (n = 1 641 225)		HDHP group (n = 156 962)	Control group (n = 1 467 758)	
Age, mean (SD), y	52.7 (6.7)	52.9 (7.0)	−0.023	52.7 (6.7)	52.9 (6.9)	−0.028
Female	73 830 (46.7)	791 364 (48.2)	−0.031	73 290 (46.7)	687 802 (46.9)	−0.003
Diabetes^b^	28 454 (18.0)	298 288 (18.2)	0.044	29 407 (18.7)	274 986 (18.7)	0
Cardiovascular disease^c^	9778 (6.2)	88 746 (5.4)		10 174 (6.5)	95 137 (6.5)	
Hypertension or hyperlipidemia^d^	119 947 (75.8)	1 254 191 (76.4)		117 381 (74.8)	1 097 634 (74.8)	
Living in neighborhoods with below-poverty levels, %^e^						
<5.0	38 107 (24.1)	430 006 (26.2)	0.062	37 825 (24.1)	361 385 (24.6)	0.056
5.0-9.9	42 764 (27.0)	452 258 (27.6)		42 479 (27.1)	408 051 (27.8)	
10.0-19.9	48 342 (30.6)	479 843 (29.2)		48 085 (30.6)	448 519 (30.6)	
≥20	28 703 (18.1)	277 422 (16.9)		28 561 (18.2)	249 599 (17.0)	
Missing	263 (0.2)	1696 (0.1)		12 (0.0)	204 (0.0)	
Living in neighborhoods with educational levels below high school, %^e^						
<15.0	112 468 (71.1)	1 190 091 (72.5)	0.088	111 743 (71.2)	1 069 721 (72.9)	0.049
15.0-24.9	30 309 (19.2)	299 086 (18.2)		30 137 (19.2)	268 160 (18.3)	
25.0-39.9	12 295 (7.8)	123 915 (7.6)		12 240 (7.8)	106 726 (7.3)	
≥40.0	2847 (1.8)	26 468 (1.6)		2833 (1.8)	22 975 (1.6)	
Missing educational level	260 (0.2)	1665 (0.1)		9 (0.0)	176 (0.0)	
Race/ethnicity^f^						
Asian	3508 (2.2)	47 149 (2.9)	0.127	3466 (2.2)	32 196 (2.2)	0.038
Black	3580 (2.3)	50 816 (3.1)		3563 (2.3)	33 391 (2.3)	
Hispanic	12 085 (7.6)	134 564 (8.2)		11 943 (7.6)	109 861 (7.5)	
Mixed	31 412 (19.9)	369 955 (22.5)		31 235 (19.9)	299 128 (20.4)	
White	107 400 (67.9)	1 037 425 (63.2)		106 749 (68.0)	993 027 (67.7)	
Missing	194 (0.1)	1316 (0.1)		6 (0.0)	154 (0.0)	
Age category, y						
40-49	53 136 (33.6)	555 950 (33.9)	0.054	52 823 (33.7)	488 731 (33.3)	0.050
50-59	74 708 (47.2)	730 576 (44.5)		74 138 (47.2)	673 347 (45.9)	
60-64	30 335 (19.2)	354 699 (21.6)		30 001 (19.1)	305 680 (20.8)	
ACG score, mean (SD)^g^	1.5 (2.3)	1.5 (2.3)	−0.018	1.5 (2.2)	1.5 (2.2)	0.007
US region						
South	76 860 (48.6)	746 850 (45.5)	0.212	76 789 (48.9)	762 632 (52.0)	0.084
West	14 917 (9.4)	204 120 (12.4)		14 904 (9.5)	148 105 (10.1)	
Midwest	54 364 (34.4)	486 910 (29.7)		54 315 (34.6)	473 900 (32.3)	
Northeast	10 955 (6.9)	195 252 (11.9)		10 954 (7.0)	83 121 (5.7)	
Missing	1083 (0.7)	8093 (0.5)				
Outpatient copayment, median (SD), $	19.3 (6.2)	16.8 (6.9)	0.383	19.3 (6.2)	18.6 (6.4)	0.107
Baseline total costs, (SD), $	10 784 (25 611)	11 127 (26 942)	−0.013	10 751 (25443)	10 529 (24 124)	0.009
Out-of-pocket spending, $						
0.00-500.00	57 763 (36.5)	689 791 (42.0)	0.171	57 192 (36.4)	535 842 (36.5)	0.066
500.01-999.99	38 205 (24.2)	422 100 (25.7)		38 016 (24.2)	368 264 (25.1)	
1000.00-2499.99	44 173 (27.9)	404 436 (24.6)		43 914 (28.0)	415 363 (28.3)	
≥2500.00	18 038 (11.4)	124 898 (7.6)		17 840 (11.4)	148 289 (10.1)	
Employer size, No. of enrollees						
0-99	101 951 (64.5)	317 329 (19.3)	1.299	100 970 (64.3)	920 475 (62.7)	0.124
100-999	49 139 (31.1)	537 972 (32.8)		48 908 (31.2)	431 698 (29.4)	
≥1000	7089 (4.5)	785 924 (47.9)		7084 (4.5)	115 585 (7.9)	

Abbreviation: HDHP, high-deductible health plan.

^a^ Closer to zero indicates greater similarity.

^b^ Based on Johns Hopkins ACG software definition of diabetes.

^c^ Based on Johns Hopkins ACG software definition of cardiovascular disease.

^d^ Based on Johns Hopkins ACG software definition of hypertension/hyperlipidemia.

^e^ Based on 2008-2012 American Community Survey data at census tract level.

^f^ Definitions available in Methods Covariates subsection.

^g^ Based on Johns Hopkins ACG Software; mean score in overall sample (members in and not in this cohort) was 0.62 to 0.82 from 2003 to 2014.

**Figure 1.**
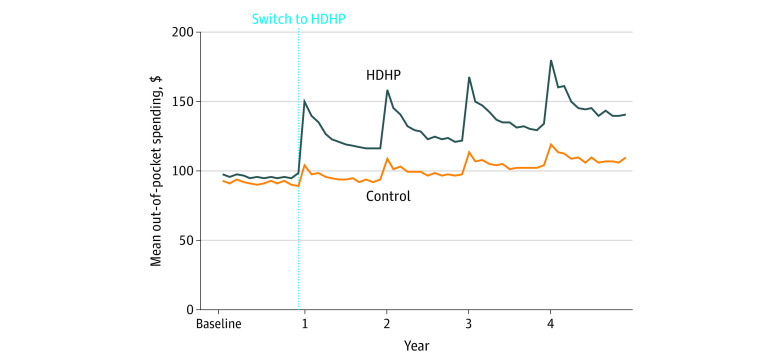
Total Out-of-Pocket Expenditures in the High-Deductible Health Plan (HDHP) Group and the Control Group

Individuals with HDHPs experienced total (medical plus pharmacy) out-of-pocket expenditure increases ranging from 25.7% (95% CI, 24.2%-27.1%) to 31.1% (95% CI, 28.3%-33.9%) per follow-up year vs baseline and relative to controls (Figure 1; eTable 5 in the Supplement). Corresponding ranges for medical expenditure increases were 41.1% (95% CI, 38.3%-43.9%) to 52.2% (95% CI, 47.2%-57.2%). In contrast, pharmacy out-of-pocket expenditures increased by only 5.3% (95% CI, 4.1%-6.5%) to 6.7% (95% CI, 6.3%-7.1%) during the same period.

**Figure 2.**
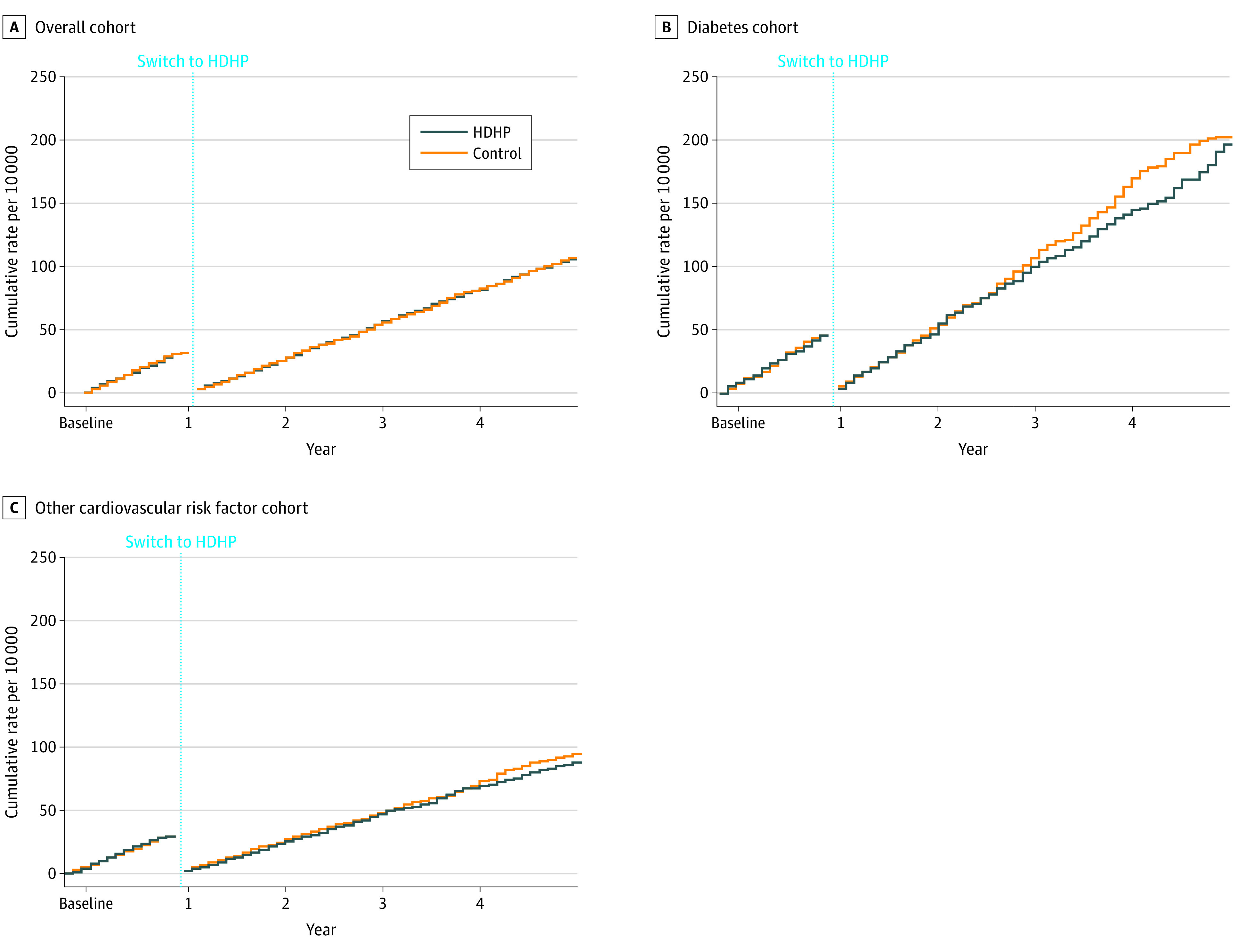
Weighted and Adjusted Cumulative Rates of First Major Cardiovascular Events in the High-Deductible Health Plan (HDHP) Group and Control Group First major cardiovascular events comprise myocardial infarction or stroke. A, Overall cohort includes patients with diabetes, cardiovascular disease, hypertension, or hyperlipidemia diagnosed before the index date (adjusted hazard ratio [aHR] for follow-up vs baseline year, 1.00; 95% CI, 0.89-1.13). B, Diabetes cohort defined using Johns Hopkins ACG software and diagnosed before the index date (aHR for follow-up vs baseline year, 0.93; 95% CI, 0.75-1.16). C, Other cardiovascular risk factor cohort comprises patients with cardiovascular disease, hypertension, or hyperlipidemia based on Johns Hopkins ACG software and diagnosed before the index date (aHR for follow-up vs baseline year, 0.93; 95% CI, 0.81-1.07). Outcome measures could occur more than once per person. We measured first events per person in both the baseline and follow-up periods, thus “resetting” each person to zero events at the beginning of the follow-up period.

At follow-up, compared with baseline, first major adverse cardiovascular events among HDHP members did not differ relative to controls (aHR, 1.00; 95% CI, 0.89-1.13) (Figure 2). Findings were similar among subgroups with diabetes (aHR, 0.93; 95% CI, 0.75-1.16) and those with other cardiovascular risk factors (aHR, 0.93; 95% CI, 0.81-1.07). The subgroup with diabetes, established cardiovascular disease, and either hypertension or hyperlipidemia had an aHR, of 0.76 (95% CI, 0.57-1.03) (eTable 7 in the Supplement). Finally, HDHP members with index dates in 2009 or after had an aHR of 1.07 (95% CI, 0.87-1.32) (eTable 7 in the Supplement).

**Figure 3.**
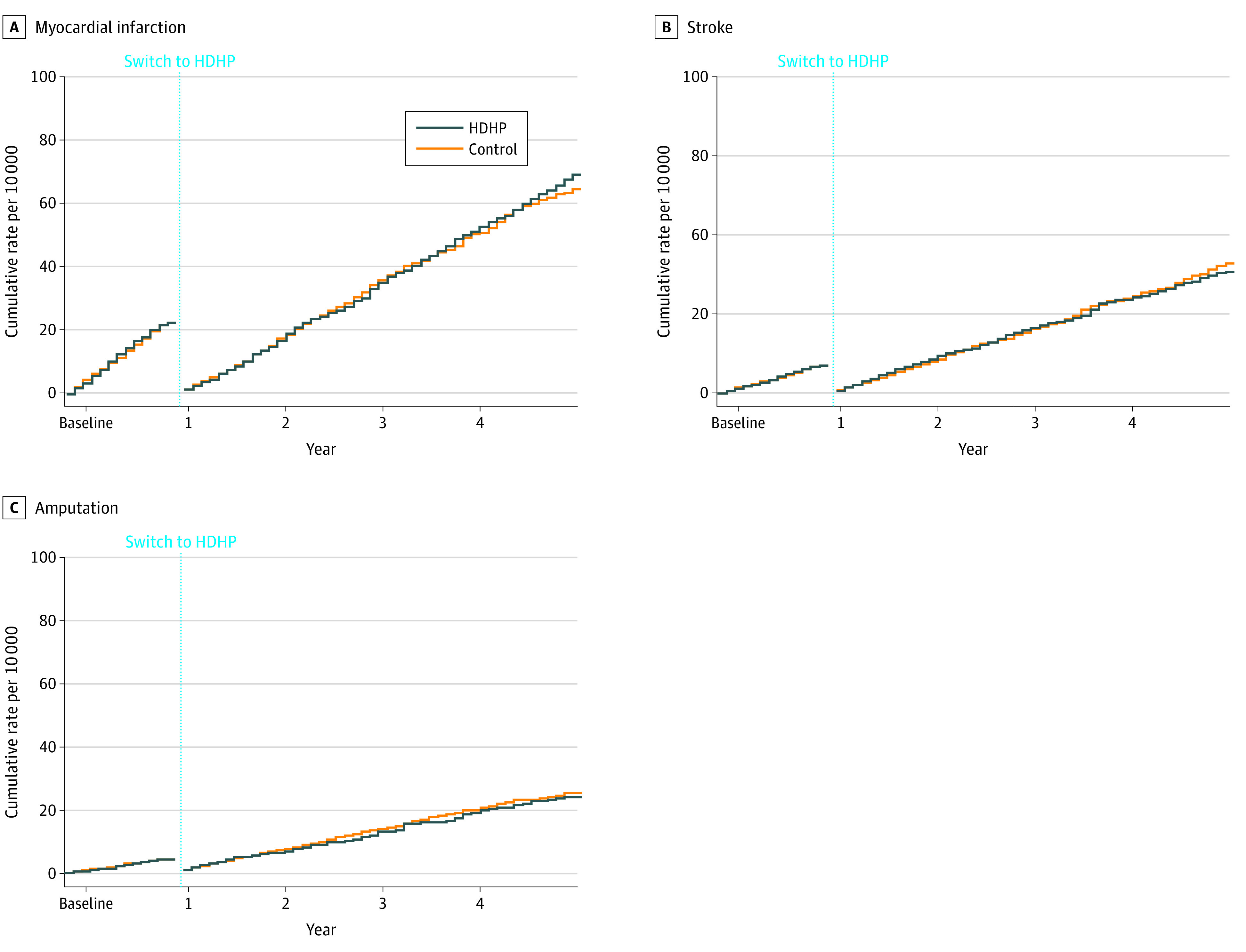
Weighted and Adjusted Cumulative Rates of First Myocardial Infarction, Stroke, and Amputation in the Overall High-Deductible Health Plan (HDHP) Cohort and Control Cohort A, Myocardial infarction defined as 3 or more days of hospitalization with a myocardial infarction diagnosis at hospital discharge (adjusted hazard ratio [aHR] for follow-up vs baseline year, 1.02; 95% CI, 0.88-1.17). B, Stroke defined as 3 or more days of hospitalization with stroke diagnosis at hospital discharge (aHR for follow-up vs baseline year, 0.99; 95% CI, 0.81-1.23). C, Amputation defined as based on the presence of billing codes for amputation procedures (aHR for follow-up vs baseline year, 0.95; 95% CI, 0.71-1.27); see Methods section for details. Outcome measures could occur more than once per person. We measured first events per person in both the baseline and follow-up periods, thus “resetting” each person to zero events at the beginning of the follow-up period.

In analyses of secondary disaggregated outcomes, HDHP members did not experience detectable differences in time to first myocardial infarction (aHR, 1.02; 95% CI, 0.88-1.17) (Figure 3), stroke (aHR, 0.99; 95% CI, 0.81-1.23), or amputation (aHR, 0.95; 95% CI, 0.71-1.27). Adding all-cause mortality to our primary composite outcome did not change interpretation of results (follow-up aHR, 0.98; 95% CI, 0.93-1.04) (eFigure 2 in the Supplement), including when such analyses were restricted to before November 2011 (follow-up aHR, 0.97; 95% CI, 0.92-1.03). Post hoc analyses revealed that HDHP members experienced approximately 2% to 6% relative reductions in the use of cardioprotective medications relative to controls during the 4 follow-up years (eTable 6 in the Supplement).

## Discussion

In the present study, HDHP members did not experience a detectable increase in major adverse cardiovascular events compared with individuals in low-deductible plans, and we can reasonably rule out hazard ratio increases above 1.13. Results were similar among subgroups with diabetes and other cardiovascular disease risk factors, although the upper limit of the 95% CIs in the subgroups was 1.16. Thus, our findings suggest that overall cost-sharing increases of approximately 25% to 30% were not associated with cardiovascular care alterations to a degree large enough to observe increases in myocardial infarction, stroke, or amputation rates during the 4 years assessed in this study. Previously reported 1.5- to 3.1-month delays in cardiovascular care among HDHP members^11^ might therefore have been clinically insignificant or might have represented reductions in discretionary care.

Our study adds important evidence about health effects associated with high cost sharing for patients and with modern insurance designs. Commentators have raised concerns that HDHPs would worsen major health outcomes,^32^ and we also hypothesized this association based on recent HDHP studies.^9,10^

Several factors might explain the discrepancy between our expectations and the observed findings. First, modern HDHPs typically include features such as low or no out-of-pocket costs for medications and preventive services. The minimal changes we detected in cardiovascular medication use and preventive services^10^ might therefore have protected HDHP members from increased adverse cardiovascular events. Second, previous research indicates that patients with low-income and high-morbidity are at greatest risk of adverse cost-sharing outcomes.^8,9,10^ Our analyses were not powered to examine these vulnerable subgroups although such individuals are more likely to be in Medicaid or to be uninsured than to have commercial insurance. Third, increased major cardiovascular events might only occur with substantially higher cost sharing. Mean deductibles in our study were approximately $2000, levels that are representative of the employer-sponsored market.^16^ Future studies could focus on plans with individual deductibles of greater than, for example, $3000, but the low prevalence of such plans^16^ raises analytic challenges. Finally, increased major cardiovascular events might only manifest after substantially longer exposure to high cost sharing. However, trends toward such patterns were not visible in our time-to-event plots.

Although our findings provide a measure of reassurance that HDHP enrollment was not associated with an appreciable increased risk of major adverse cardiovascular outcomes during 4 follow-up years, we note several caveats and recommendations. Most importantly, policymakers and employers should remain cautious in promoting HDHPs among low-income and other vulnerable patients given the potential for adverse financial and health outcomes that this study did not address. Research should also extend follow-up time to better assess long-term outcomes and should examine whether people with HDHPs ultimately require more intensive workups and more advanced treatments for cardiovascular events.

Future research should also compare major adverse cardiovascular events among HDHP members who do or who do not have value-based insurance features,^13,33^ such as exempting medications from deductible payments. The US Department of the Treasury recently allowed health savings account HDHPs to exempt secondary preventive medications (such as diabetes and cardioprotective drugs) from the annual deductible level.^34^

Our study included several features to minimize bias, including restricting the study to employers that do not allow enrollee choice of deductible level and matching to balance key employer- and individual-level characteristics. We also adjusted outcomes for any preintervention differences between study groups.

### Limitations

Our analyses were observational and therefore at risk for bias from unmeasured confounders. Although we had exact deductible amounts of most of the smaller employers, we imputed deductible levels from claims for almost all large employers. However, we do not believe that the imputation biased our results because of its high sensitivity and specificity (eTable 1 in the Supplement). We did not have access to health savings account balances among the minority of HDHP members in our study who had such accounts, but excluding these members from the overall cohort did not appreciably change the findings (eTable 8 in the Supplement). Our study findings are not generalizable to individuals with uncommonly high deductibles, newly insured patients, or people without identified cardiovascular risk factors. We also acknowledge that although our design reduced selection into deductible levels at the individual level, employers might have taken employees’ health status into account when determining whether to exclusively offer HDHPs. To account for this potential source of bias, we matched study groups on employer-level characteristics. Although we studied patients enrolling in HDHPs over a broad period, results among later-enrolling members did not differ statistically from those of the overall cohort. Nevertheless, future research should assess outcomes of more recent cohorts given continuing increases in deductible levels. Finally, approximately 20% of enrollees had 4 years of follow-up time; if members with this longer-term follow-up are not representative of the overall cohort, our results could be biased.

## Conclusions

Mandated enrollment in HDHPs was not associated with a significantly increased risk of major adverse cardiovascular events during 4 years of follow-up in the present study. Future studies should include longer follow-up, rigorously examine effects of value-based medication cost-sharing arrangements within HDHPs,^13,33^ and assess outcomes among low-income HDHP members.

## Notes

Supplement. **eAppendix.** Methods and Results **eTable 1.** Validation of Deductible Imputation Algorithm, Stratified by Employer Size **eFigure 1.** Study Design Showing Example Members of the High-Deductible Health Plan Group and Matched Control Group **eTable 2.** Baseline Characteristics of the Subgroup With Diabetes Before and After Matching **eTable 3.** Baseline Characteristics of the Subgroup With Established Cardiovascular Disease, Hypertension, or Hyperlipidemia Before and After Matching **eTable 4.** Billing Codes Used to Create Measures of Myocardial Infarction, Stroke, and Extremity Amputation **eTable 5.** Out-of-Pocket Total (Medical and Pharmacy), Medical, and Pharmacy Expenditure Changes in the HDHP and Control Groups in Follow-up Years 1 to 4 Compared With the Baseline Year **eTable 6.** Cardioprotective Medication Fills per Year in the High-Deductible Health Plan and Control Groups in Follow-up Years 1-4 Compared with the Baseline Year **eTable 7.** Adjusted Hazard Ratios for Sensitivity Analyses Examining a Very High-Risk Subgroup and a Cohort With Index Dates in 2009 and After **eTable 8.** Adjusted Hazard Ratios for Sensitivity Analyses That Exclude HDHP Members With Health Savings Accounts or Health Reimbursement Arrangements **eFigure 2.** Weighted and Adjusted Cumulative Rates of First Major Cardiovascular Events and All-Cause Mortality in the HDHP and Control Groups **eReferences**
