# Supplementary material for: Association Between Switching to a High-Deductible Health Plan and Major Cardiovascular Outcomes
Source: JAMA Netw Open. 2020 Jul 24;3(7):e208939. doi: 10.1001/jamanetworkopen.2020.8939 (PMC7382004; doi:10.1001/jamanetworkopen.2020.8939)
Supplement: Supplement. — eAppendix. Methods and Results eTable 1. Validation of Deductible Imputation Algorithm, Stratified by Employer Size eFigure 1. Study Design Showing Example Members of the High-Deductible Health Plan Group and Matched Control Group eTable 2. Baseline Characteristics of the Subgroup With Diabetes Before and After Matching eTable 3. Baseline Characteristics of the Subgroup With Established Cardiovascular Disease, Hypertension, or Hyperlipidemia Before and After Matching eTable 4. Billing Codes Used to Create Measures of Myocardial Infarction, Stroke, and Extremity Amputation eTable 5. Out-of-Pocket Total (Medical and Pharmacy), Medical, and Pharmacy Expenditure Changes in the HDHP and Control Groups in Follow-up Years 1 to 4 Compared With the Baseline Year eTable 6. Cardioprotective Medication Fills per Year in the High-Deductible Health Plan and Control Groups in Follow-up Years 1-4 Compared with the Baseline Year eTable 7. Adjusted Hazard Ratios for Sensitivity Analyses Examining a Very High-Risk Subgroup and a Cohort With Index Dates in 2009 and After eTable 8. Adjusted Hazard Ratios for Sensitivity Analyses That Exclude HDHP Members With Health Savings Accounts or Health Reimbursement Arrangements eFigure 2. Weighted and Adjusted Cumulative Rates of First Major Cardiovascular Events and All-Cause Mortality in the HDHP and Control Groups eReferences [file jamanetwopen-3-e208939-s001.pdf]

Supplementary Online Content

Wharam JF, Wallace J, Zhang F, et al. Association between switching to a high-deductible health plan and major cardiovascular outcomes. *JAMA Netw Open*. 2020;3(7):e208939. doi:10.1001/jamanetworkopen.2020.8939

eAppendix. Methods and Results
eTable 1. Validation of Deductible Imputation Algorithm, Stratified by Employer Size
eFigure 1. Study Design Showing Example Members of the High-Deductible Health Plan Group and Matched Control Group
eTable 2. Baseline Characteristics of the Subgroup With Diabetes Before and After Matching
eTable 3. Baseline Characteristics of the Subgroup With Established Cardiovascular Disease, Hypertension, or Hyperlipidemia Before and After Matching
eTable 4. Billing Codes Used to Create Measures of Myocardial Infarction, Stroke, and Extremity Amputation
eTable 5. Out-of-Pocket Total (Medical and Pharmacy), Medical, and Pharmacy Expenditure Changes in the HDHP and Control Groups in Follow-up Years 1 to 4 Compared With the Baseline Year
eTable 6. Cardioprotective Medication Fills per Year in the High-Deductible Health Plan and Control Groups in Follow-up Years 1-4 Compared with the Baseline Year
eTable 7. Adjusted Hazard Ratios for Sensitivity Analyses Examining a Very High-Risk Subgroup and a Cohort With Index Dates in 2009 and After
eTable 8. Adjusted Hazard Ratios for Sensitivity Analyses That Exclude HDHP Members With Health Savings Accounts or Health Reimbursement Arrangements
eFigure 2. Weighted and Adjusted Cumulative Rates of First Major Cardiovascular Events and All-Cause Mortality in the HDHP and Control Groups
eReferences

This supplementary material has been provided by the authors to give readers additional information about their work.

© 2020 Wharam JF et al. *JAMA Network Open*.

eAppendix. Methods and Results

I. eMethods

A. Study Group Construction and Individual-level Deductible Imputation Algorithm

To determine employer deductible levels, we used a benefits type variable that we had for most smaller employers (with approximately 100 or fewer employees). For larger employers, we took advantage of the fact that health insurance claims data are the most accurate source for assessing out-of-pocket obligations among patients who utilize health services. Our claims data contained an in-network/out-of-network individual deductible payment field. For patients who use expensive or frequent services, the sum of their yearly deductible payments adds up to clearly identifiable exact amounts such as $500.00, $1000.00, $2000.00, etc. When even several members have these same amounts, it provides strong evidence that the employer offered such an annual deductible level. It is also possible to detect employers that offer choices of deductible levels when multiple employees have deductibles at two or more levels, such as 20 employees with an exact annual amount of $1000.00 and 12 employees with $500.00. For employer accounts with at least 10 enrollees, we therefore summed each member's in-network (individual-level) deductible payments and number of claims over the enrollment year and assessed other key characteristics such as percentage with Health Savings Accounts. We randomly selected half of the employer account data set that contained both our calculated employer characteristics (independent variables, below) and actual annual deductible levels from the benefits table (dependent variable, after categorization; below). We then used a multinomial logistic model that predicted the 4-level outcome of individual-level deductible ≤$500/$500-$999/$1000-$2499/≥$2500 (again, dependent variable) based on multiple aggregate employer characteristics (independent variables) such as the percentage with Health Savings Accounts and Health Reimbursement Arrangements, the deductible payment per employer in the 75 percentile of payments, the percentage of employees reaching exact deductible levels or with deductible payments but not reaching an exact deductible level, the employer account size, the percentage of enrollees per account with summed whole dollar annual deductible amounts (from claims data) between $0 to <$100, ≥$100 to ≤$500, >$500 to <$1000, ≥$1000 to <$2500, ≥$2500, etc.

The statistical model was as follows:

$$\text{Logit}(\text{Pr}=Y_i) = \beta_0 + \sum \beta_k X_{ki}$$

Where:

Y_i = dependent variable (4-level deductible category)

X_{ki}= k^{th} characteristics for i^{th} employer

$\beta_{0=}$ intercept

$\beta_{k=}$ coefficient for k^{th} characteristic

The SAS code we used to implement this model was:

```
proc logistic data=csn_impute_PLUS_to_be_imputed descending;
class
        d_wusd1perc_0_100_cat d_wusd1perc_100_500_cat d_wusd1perc_500_1000_cat
        d_wusd1perc_1000_2500_cat d_wusd1perc_ge2500_cat
        d_wusd2perc_0_100_cat d_wusd2perc_100_500_cat d_wusd2perc_500_1000_cat
        d_wusd2perc_1000_2500_cat d_wusd2perc_ge2500_cat
        d_wusd3perc_0_100_cat d_wusd3perc_100_500_cat d_wusd3perc_500_1000_cat
        d_wusd3perc_1000_2500_cat d_wusd3perc_ge2500_cat
        d_wusd4perc_0_100_cat d_wusd4perc_100_500_cat d_wusd4perc_500_1000_cat
        d_wusd4perc_1000_2500_cat d_wusd4perc_ge2500_cat;

model real_dduct_cat =
        pyr sampletot hsa_cnt_over_total cdhp_cnt_over_total perc_grp2 perc_grp3 perc_grp4 perc_grp5
        d_wusd1perc_0_100_cat d_wusd1perc_100_500_cat d_wusd1perc_500_1000_cat
        d_wusd1perc_1000_2500_cat d_wusd1perc_ge2500_cat d_wusd2perc_0_100_cat
        d_wusd2perc_100_500_cat d_wusd2perc_500_1000_cat d_wusd2perc_1000_2500_cat
        d_wusd2perc_ge2500_cat d_wusd3perc_0_100_cat d_wusd3perc_100_500_cat
        d_wusd3perc_500_1000_cat d_wusd3perc_1000_2500_cat d_wusd3perc_ge2500_cat
```

© 2020 Wharam JF et al. *JAMA Network Open*.

```
        d_wusd4perc_0_100_cat d_wusd4perc_100_500_cat d_wusd4perc_500_1000_cat
        d_wusd4perc_1000_2500_cat d_wusd4perc_ge2500_cat
        p75_0_100_dduct p75_100_500_dduct p75_500_1000_dduct p75_1000_2500_dduct p75_gt2500_dduct
output out=prob_of_dduct_cat&IOS. p=p_dduct_cat predprobs=i;
run;
```

Further explanation of this code is below. Note that all values described are calculated over the benefit year per employer account, and a given employer account could be present for multiple years.

- csn_impute_PLUS_to_be_imputed = name of dataset that contains, at the employer account and benefit year level, accounts with missing deductible levels as well as a random half of the accounts that have actual deductible levels. The other random half is also present in the dataset but with actual deductible levels "hidden" so that they can later be used to validate the predictive algorithm.
- real_dduct_cat = dependent variable; category of actual deductible level from the gold standard source (<=$500, $500-$999, $1000-$2499, ≥$2500)
- pyr = benefit year of account's information and tied to the calendar year. An employer could have multiple benefit years represented in separate records per account-benefit year.
- sampletot = total enrollees per account during the benefit year
- hsa_cnt_over_total = percent of members per account listed as having a health savings account
- cdhp_cnt_over_total = percent of members per account listed as having a health savings account or health reimbursement arrangement
- perc_grp1. Percentage of enrollees per employer-year who have claims but $0 deductible amounts for all annual claims.
- perc_grp2. Percentage of enrollees per employer-year who have reached their annual deductible, evidenced by the sum of their deductible payments ending in $*0.00. Members must have at least one month after the month of the $*0.00 summation where the deductible field is blank, and all subsequent months must have blank deductible fields, indicating that the member reached his or her annual deductible amount.
- perc_grp3. Percentage of enrollees per employer-year who have an annual deductible amount that does not end in $*0.00.
- perc_grp4. Percentage of enrollees per employer-year who have enrollment during the benefit year where all months show no evidence of utilization (no health insurance claims).
- perc_grp5. Percentage of enrollees per employer-year who might have reached their deductible, as evidenced by having the last month of enrollment of the benefit year with a summed annual deductible amount that ends in $*0.00.
- d_wusd1perc_0_100_cat, d_wusd1perc_100_500_cat, d_wusd1perc_500_1000_cat, d_wusd1perc_1000_2500_cat d_wusd1perc_ge2500_cat. Category of percentage of enrollees with an employer's most common whole number annual individual deductible payment total (e.g. dollar amount ending in 0.00) per employee that is $0 to <$100, ≥$100 to ≤$500, >$500 to <$1000, ≥$1000 to <$2500, and ≥$2500, respectively.
- d_wusd2perc_0_100_cat, d_wusd2perc_100_500_cat, d_wusd2perc_500_1000_cat, d_wusd2perc_1000_2500_cat d_wusd2perc_ge2500_cat. Category of percentage of enrollees with an employer's second most common whole number annual individual deductible payment total (e.g. dollar amount ending in 0.00) per employee that is $0 to <$100, ≥$100 to ≤$500, >$500 to <$1000, ≥$1000 to <$2500, and ≥$2500, respectively.
- d_wusd3perc_0_100_cat, d_wusd3perc_100_500_cat, d_wusd3perc_500_1000_cat, d_wusd3perc_1000_2500_cat d_wusd3perc_ge2500_cat. Category of percentage of enrollees with an employer's third most common whole number annual individual deductible payment total (e.g. dollar amount ending in 0.00) per employee that is $0 to <$100, ≥$100 to ≤$500, >$500 to <$1000, ≥$1000 to <$2500, and ≥$2500, respectively.
- d_wusd4perc_0_100_cat, d_wusd4perc_100_500_cat, d_wusd4perc_500_1000_cat, d_wusd4perc_1000_2500_cat d_wusd4perc_ge2500_cat. Category of percentage of enrollees with an employer's fourth most common whole number annual individual deductible payment total (e.g. dollar amount ending in 0.00) per employee that is $0 to <$100, ≥$100 to ≤$500, >$500 to <$1000, ≥$1000 to <$2500, and ≥$2500, respectively.

© 2020 Wharam JF et al. *JAMA Network Open*.

- p75_0_100_dduct p75_100_500_dduct p75_500_1000_dduct p75_1000_2500_dduct p75_gt2500_dduct. Category of 75th percentile of deductible payments per employer benefit year, categorized as $0 to <$100, ≥$100 to ≤$500, >$500 to <$1000, ≥$1000 to <$2500, and ≥$2500, respectively.

This predictive model output the probability that employers had deductibles in the four categories (summing to 1.0) and we assigned the employer to the level that had the highest probability. We overwrote this assignment with the most common whole number deductible amount per year if it was not zero, and with the second most common whole number deductible amount if the most common amount was zero and at least 10 members had the value of the second most common whole number deductible amount. If an employer had members with both enrollment and evidence of utilization, but never had any amounts in the deductible field, we assigned that employer to <$500 deductible level. If an employer had only members that reached a whole number annual deductible amount such as $1000.00 or $2000.00, we assigned the most common deductible amount as the employer's deductible if that amount was greater than or equal to $1000 and to the 95% percentile value if that number was less than $1000. If at least 99% of employees had Health Savings Accounts or Health Reimbursement Arrangements, we also overwrote any previous assignment to classify the employer as a high-deductible employer. We assigned employers to have a choice between deductible levels of $1000 to $2499 and ≥$2500 when both were common and one accounted for at least 85% of $1000-$2499 or ≥$2500 deductible levels reached per employer. If we detected employers that had sufficient enrollees with whole number deductible levels both above and below $1000 (e.g. $250.00 and $1500.00), we assigned the employers' category as "choice," applying a similar 85% rule. Finally, for any employer that had gold standard deductible level information in our benefits file, we overwrote any previous imputed deductible level.

Our file that contains actual deductible amounts per employer covers the "small employer" segment of the insurer's business, a segment that generally includes employers with fewer than 100 or so enrollees. However, it does include a modest number of employers with more than 100 enrollees, even up to approximately 1000 enrollees. The histograms below, where the x-axis represents employer size and the y-axis shows the percentage of employers that are that size, demonstrate the distribution of employer sizes. The second plot "magnifies" the y-axis to demonstrate the smaller number of large employers.

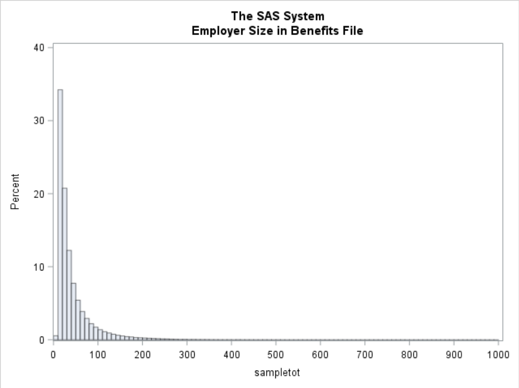

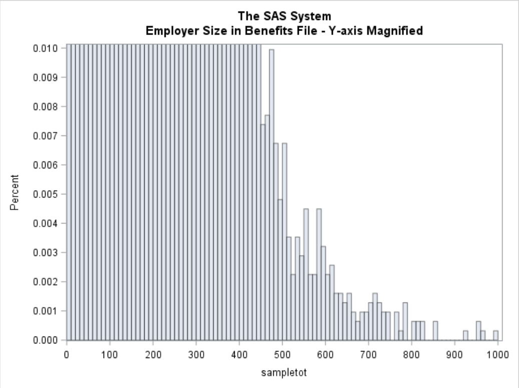

To demonstrate the robustness of our imputation algorithm, and its predictive value as employer size increases (given that we do not have benefits information on most large employers), we took advantage of the fact that although this file mostly covers employers with 100 enrollees or fewer, there is some overlap with larger employers (i.e., those with ~100 to 1000 enrollees). A random half of our imputation sample had the actual deductible levels of employers of all sizes "hidden" from the imputation. Thus, this random half included a modest number of employers with 75 to 1000 enrollees. We tested the sensitivity and specificity of the imputation in this overlap zone, categorizing employer sizes as 75-100, 101-400, 401-700, and 701-1000 enrollees (Exhibit 1). At employers with 75-100 enrollees, we found sensitivity of 95.4% and specificity of 98.3% (Exhibit 1a). Sensitivity and specificity increased across employer size to 100%, and Exhibits 1b-1d display these for employers of sizes 101-400, 401-700, and 701-1000.

We used an employer ID and an algorithm that determined linked employer subaccounts to identify an employer's subaccounts per benefit year, and removed benefit years when employers offered both low and high deductible levels.

Rationale for High-Deductible Cutoffs: When Health Savings Account-eligible high-deductible health plans came to market in 2005-2006, the Internal Revenue Service set the minimum deductible level for qualifying high-deductible health plans at $1050 (which could be adjusted upward for inflation annually). The range of this minimum deductible during our study period was $1050-$1250. For these reasons, we defined high-deductible health plans as annual individual deductibles of at least $1000 (otherwise some health savings account plans would be excluded). In addition, choosing this

© 2020 Wharam JF et al. *JAMA Network Open*.

cutoff (as opposed to, e.g., $2000) improves the sensitivity and specificity of the imputation because this is common deductible level and more enrollees per employer meet this threshold. This cutoff is also a “real-world” deductible minimum that allows the most generalizable results. It should also be noted that $1000 was the *minimum* annual deductible level we included and not the mean deductible level. We cannot precisely calculate the mean deductible level of the high-deductible health plan group, but we estimate, using the most common non-zero deductible levels per employer account, an approximate mean deductible of $1900. We defined traditional plans as having deductible levels of ≤$500 after determining that a threshold of ≤$250 would lead to an inadequate sample size for the control group. Again, the mean deductible level of the control group members would be lower than $500.

Our high-deductible health plan group comprised the enrollment years of employers that had a year-on-year transition from low- to high-deductible coverage (i.e., from $500 or less to $1000 or more). Some members had multiple eligible index dates (e.g., multiple low-to-low deductible years or both low-to-low and low-to-high deductible years). In the cases of members with both low-to-low and low-to-high deductible years, we randomly assigned enrollees to the high-deductible health plan or control pool. Then, for members assigned to the control pool that had multiple low-to-low deductible spans, we randomly selected one of their potential index dates (and their corresponding before-after enrollment years).

There were 1,799,404 members with 3,908,743 unique potential patient index date combinations within the overall cohort. Among them, 1,600,531 members had only low-to-low deductible years, 117,217 members had only low-to-high deductible enrollment, and 81,656 members had both low-to-low and low-to-high deductible enrollment. Among those 81,656 members, we first randomly assigned members with statistical “coin flip” to a study group, resulting in 40,962 members for whom we randomly assigned their low-to-high deductible plan switch date as their index date. A very small number of these members had more than one low-to-high deductible plan switch date, so we also randomly chose one of those. For the members remaining from the initial 81,656 sample, we randomly assigned one of their low-to-low deductible plan switches as their index date. Next, among the 1,600,531 members with multiple potential low-to-low deductible index dates, we randomly selected one of the index dates. The final cohort included 158,179 members who underwent a switch from a low-deductible health plan to a high-deductible health plan and 1,641,225 members who remained in a low-deductible health plan throughout the baseline and follow-up periods.

Population. Our study population included people with risk factors for cardiovascular disease at baseline so we could observe the occurrence of major adverse cardiovascular events at baseline and over 4 follow-up years. These individuals were commercially insured members in a large national health insurance database enrolled between 1/1/2003-12/31/2014. This database includes approximately 48 million members with commercial insurance along with their enrollment information and all medical, pharmacy, and hospitalization claims. We included only members with employer-sponsored insurance.

We considered an insurance plan to have a low deductible if the annual deductible was ≤$500, and we considered a plan to have a high-deductible if the annual amount was ≥$1000. For smaller employers, we determined the deductible amount from a benefits table obtained from the health insurer. This benefits table mostly included employers with fewer than 100 employees and dependents, but also included a modest number of larger employers (see above). For employers not represented in the insurance benefits table (mostly large employers), we imputed deductible amounts from actual out-of-pocket spending by individuals who utilized health services, using an algorithm with a sensitivity of 98-100% and specificity of 95-100% depending on employer size (eTable1).

The individuals in this study were not able to choose a low-deductible level versus a high-deductible level, because each employer provided only one level of deductible each year. Some employers offered a low-deductible plan throughout the study, and others that had offered a low-deductible plan during an earlier time switched to a high-deductible plan for the remainder of the study.

We defined the index date for employers who switched to high-deductible plans as the beginning of the month when the switch occurred. We defined the index date for employers who did not switch plans as the beginning of the month when their yearly account was renewed. If an employer had multiple potential index dates, e.g., 5 continuous years with a low-deductible plan or 4 years in a low-deductible plan followed by 1 year in a high-deductible plan, we randomly selected 1 index date. People entered the study at different times because their employers had different index dates. For all individuals in the study, the beginning of study time (time zero) was 12 months before their employers’ index date and we defined this 12-month period as the baseline year (eFigure 1). The employer’s index date was the beginning of the follow-up period. For each individual, we measured months from the beginning of the baseline period to the first outcome in the baseline period, and we measured months from the index date to the first outcome in the follow-up period.

© 2020 Wharam JF et al. *JAMA Network Open*.

Individuals were eligible for the study if their employers were present in the database for at least 1 year before and 1 month after the index date. We then restricted to people age 40-64 because cardiovascular complications are rare before age 40. We further restricted to people who were continuously enrolled for at least 1 year before and at least 1 month after the index date. Among individuals eligible for both the high-deductible and control group, we randomly assigned individuals to one or the other. If they then also had multiple potential index dates, we randomly selected one index date. Our pre-match sample included 158,179 individuals with cardiovascular risk factors whose employers switched to high-deductible plans and 1,641,225 with the same risk factors whose employers kept low-deductible plans (Table 1).

Both high- and low-deductible plans generally cover medications, secondary preventing screening, and preventive primary care visits at low or no out-of-pocket cost. However, high-deductible health plan members on average must pay substantially higher amounts than low-deductible members for specialist care, diagnostic tests, and surgical procedures.[1]

B. Details and Justification of Study Design

Our study design and matching sought to approximate fully blocked randomization, i.e., stratification by key population characteristics then randomization within those strata. eFigure 1 demonstrates our before-after comparison between matched groups study design. In constructing our design and matching approach, we were influenced by the work of Thomas Cook and colleagues[2,3] and Gary King and colleagues[4-6] regarding optimal matching approaches for before-after with control group studies. Four key elements of our study design attempt to reduce selection bias: (1) studying only employers that *mandate* a given deductible level (2) balancing key *employer* characteristics given that employers self-select into insurance types, (3) balancing key *person-level* characteristics given that these closely influence outcome measures, and (4) attempting to balance *unmeasured* characteristics using methods validated by Thomas Cook and colleagues that we describe below.[2,3]

(1) The primary design feature we use to reduce bias is to isolate our analyses to employers that *mandate* a given deductible level so that enrollees at those employers experience an *exogenous* assignment to deductible level. To the extent that this exogenous assignment has no influence on the likelihood of outcomes except through the intervention, our design can approximate a person-level randomization of patients to study groups.

(2) However, we recognize the importance of employer-level factors given that employers “self-select” into insurance types. If such selection ultimately correlates with unmeasured patient-level factors that influence our outcome measures, an effect that we believe is likely to be minimal, reliance on the employer-mandated design feature alone could lead to biased results. Thus, we place a high priority in our matching approach on enforcing balance between *similar employer types.* We accomplish this by matching on employer propensity score tertile of propensity to switch to high-deductible health plans. We created tertiles of propensity score (rather than, for example, quintiles or a binary measure) on the general principle that we wanted enough categories to ensure that employers would match to similar employers, but not so many categories that lack of common support would excessively reduce sample size. Our approach of creating employer and member propensity tertiles (below) led to good balance of measured employer and member characteristics. To construct the employer propensity score, we included characteristics that predict high-deductible health plan switches, such as pre-switch spending levels and trends as well as employee demographics (see Section I.C).

(3) Within employer tertiles of propensity to switch, we required balance of key *member-level* characteristics. Our exact match therefore includes member propensity score tertile, predicting the person-level characteristics correlated with being switched to high-deductible health plans. As with the employer propensity score, we created tertiles of member propensity to have enough categories to ensure that members would match to similar members, but not so many categories that lack of common support would excessively reduce sample size. In summary, our approach attempts to balance similar types of people with cardiovascular risk factors from similar types of employers.

(4) However, the above 3 approaches could still leave imbalance on unmeasured characteristics that influence outcomes. To attempt to balance unmeasured characteristics in controlled before-after designs that may include selection bias, we follow the recommendations of Thomas Cook and colleagues.[2,3] These investigators have conducted “within-study comparison studies” in which institutions (with nested study subjects) are randomized to (1) being subject to a natural experiment where institutions can *self-select* into the intervention group (“group 1a”) or *self-select* into the control group (“group 1b”) or (2) being subject to a *second* randomization into an intervention group (“group 2a”) and a control group (“group 2b”). The “group 2” randomized controlled trial provides the gold standard

© 2020 Wharam JF et al. *JAMA Network Open*.

effect estimate against which multiple matching approaches and subsequent effect estimates in the “group 1” natural experiment arm can be compared. This line of work has emphasized the importance of matching on the “pretest” measure, i.e., the rate and/or timing of outcome measures before the intervention date. Overall, the investigators have concluded, across 3 within-study comparison studies, that matching on both the baseline outcome and available covariates consistently led to effect estimates that closely approximated the “group 2” gold standard randomized controlled trial results.[2,3]

C. Coarsened Exact Matching Description, Stata Coding, and Component Variables

We conducted an observational, longitudinal survival analysis study by comparing matched groups of individuals. The intervention group consisted of individuals who were in low-deductible insurance plans for 1 year and then were switched to high-deductible plans for an additional 1 month to 4 years. The control group consisted of matched individuals who remained in low-deductible plans throughout the study (eFigure 1). We matched based on the year of the index date; the propensity of the employer to mandate high-deductible insurance (component variables described below) and the propensity of individuals to work for such employers (component variables described below);[7,8] diabetes, cardiovascular disease, hypertension, or hyperlipidemia detected during or before the baseline year (yes/no); baseline occurrence of stroke, myocardial infarction, or amputation (yes/no); and follow-up duration category (years of follow up). Our reason for including baseline indicators of outcome measures (e.g., occurrence of stroke, myocardial infarction, or amputation) was to balance the future population-level risk of these events during the follow-up period.

We used coarsened exact matching[4,5,9] to match the study groups. Coarsened exact matching is a straightforward approach to balancing certain characteristics of study groups and is only a slight modification of exact matching. In exact matching, investigators determine population characteristics that they believe should be balanced between study groups. The exact matching process chooses control group members who have the exact same characteristics as intervention group members (for example, the same gender, age, and income). “Coarsening” denotes that investigators have leeway, based on their understanding of the clinical or research situation, to categorize a given matching variable into the number or type of categories that are most clinically meaningful or relevant to the research question (e.g., categorize age by 5 year versus 10 year intervals or by other meaningful strata such as 20-39, 40-49, 50-64, etc). Coarsened exact matching is then simply an exact match on the selected variables including by their investigator-defined categories.

Because the percentages of subjects with the exact same characteristics will differ between the intervention and control groups, coarsened exact matching software packages generate simple weights so that control members in the stratum represent the same percentage of the control group as intervention members in the stratum represent of the intervention group. For example, if there are 10 intervention group members who are female, high income, and residing in predominantly black neighborhoods, and they represent 2% of the intervention group, and there are 50 control group members with these same characteristics yet they represent 4% of the intervention group population, the exact matching algorithm would assign the control group members in this bin weights of 0.5 (2%/4%). This effectively “shrinks” this overrepresented control group bin from 50 to 25 members. The following is an example from our data showing a single very small stratum where the Stata coarsened exact matching algorithm upweighted the 3 controls relative to the 2 high-deductible members because the particular strata of characteristics is underrepresented in the control pool:

Observation #	Coarsened Exact Match Strata Number	De-identified Member ID	HDHP Group	Coarsened Exact Match Weights
71219	14	2143080659	0	5.50593
119039	14	3262715568	1	1.00000
182213	14	5325447707	0	5.50593
198330	14	2344191836	0	5.50593
231020	14	6484083692	1	1.00000

The authors of the approach describe the matching steps in the Stata algorithm as follows: “1. Begin with the covariates X and make a copy, which we denote X^*; 2. Coarsen X^* according to user-defined cutpoints, or [the coarsened exact matching software’s] automatic binning algorithm.; 3. Create one stratum per unique observation of X^* and place each observation in a stratum.; 4. Assign these strata to the original data, X and drop any observation whose stratum does not contain at least one treated and one control unit.”[10]

Coarsened exact matching tries to mimic stratification by population characteristics and then randomization within the defined strata (i.e., fully blocked randomization). Proponents cite numerous advantages of coarsened exact matching over other approaches such as propensity score matching, matching based on Mahalanobis distance, nearest neighbor

© 2020 Wharam JF et al. *JAMA Network Open*.

matching, and optimal matching. These advantages include better balance, lower root mean squared error, less model dependence, being robust to measurement error, ease of implementation, and compatibility with any subsequent statistical estimation model.[4-6]

Coarsened exact matching typically can only include a limited number of stratifications, even in large samples. The great challenge of matching is determining which combination of variables, out of a nearly infinite set, should be included in the match. King's work recommends an *a priori*, "clinically informed" approach to matching as would be used in a blocked randomization. Section I.B above includes rationale for our general matching approach, and below we include the Stata coarsened exact match code and details of the component matching variables:

Stata coarsened exact match code:

```
cem r_pscore3cat1 r_pscore3cat2 m_pscore3cat1 m_pscore3cat2 b_flag_149 b_flag_150 b_flag_151 index_yr
dm_ind cvd_ind hyper_ind follow_duration_cat,treatment(hdhp)
saveold all_suv_1
```

(1) r_pscore3cat1 r_pscore3cat2 = Indicators of being in a tertile category of employer propensity to mandate a switch from low- to high-deductible health insurance (classified into tertiles). This score is created from a logistic regression with the dependent variable being whether the employer mandated a switch to high-deductible insurance or mandated continuation in a low-deductible plan (0/1) and a set of predictor variables explained below. The SAS code for this regression model is as follows:

```
proc logistic data=empsty1 descending;
   model hdhp= empsize2-empsize3 agep2-agep6 pfemale racep2-racep5 edup2-edup4 povp2-
        povp4
          stdcst_int stdcst_trd emp_acg  median_copay regp2-regp4 indexmon ;
   output out=propensty prob=pscore_sty;
         ods output ParameterEstimates=param;
         ods output oddsratios=OR;
run;
```

The independent variables are constructed during the baseline period (please see manuscript for definitions of categorical variables) and include:

- empsize2-empsize3 = employer size, measured as number of subscribers and dependents per employer (1-99 in the reference, 100-999, 1000+);
- agep2-agep6 = proportion of enrollees in age category (proportion < age 20 is the reference, 20-29, 30-39, 40-49, 50-64, 65 and above);
- pfemale = proportion of women;
- racep2-racep5 = proportion of enrollees in race/ethnicity category (proportion from predominantly white neighborhood is the reference)
- edup2-edup4 = proportion of enrollees in neighborhood education category (proportion from the highest education level is the reference);
- povp2-povp4 = proportion of enrollees in neighborhood poverty category (proportion from lowest poverty level is the reference);
- stdcst_int = employer total standardized cost level at the beginning of baseline;
- stdcst_trd = employer total standardized cost trend during the baseline period;
- emp_acg = mean enrollee ACG morbidity score among patients with full baseline year enrollment;
- median_copay = median employer baseline outpatient copay for outpatient primary care visits;
- regp2-regp4 = proportion of enrollees in four U.S. regions (proportion from the West is the reference, South, Midwest, and Northeast);
- indexmon= calendar index month ("anniversary month") when high-deductible group employers mandated a switch from low-deductible to high-deductible plans; or the corresponding assigned anniversary month for employers that remained in low-deductible plans.

© 2020 Wharam JF et al. *JAMA Network Open*.

(2) m_pscore3cat1 m_pscore3cat2 = Indicators of being in a tertile category of member propensity to undergo a mandated switch from low- to high-deductible health insurance. Similar to the employer propensity score, this is created from a logistic regression with the dependent variable being whether the member experienced an employer-mandated switch to high-deductible insurance or an employer-mandated continuation in low-deductible insurance (0/1). The predictor variables are listed and explained below. The SAS code for this regression model is as follows:

```
proc logistic data=psmdat descending;
model hdhp=
      emptot  indexmon  regc2-regc4
  age_c4049 age_c5059 boopcat mem_std_cst1 mem_std_cst2 mem_std_cst3 emp_cost_r1
        emp_cost_r2 emp_cost_r3;
   ods output ParameterEstimates=param;
   ods output oddsratios=OR;
   output out=propen_all_niddk prob=pscore;
run;
```

We constructed the dependent variables, described below, during the baseline period (please see manuscript for definitions of categorical variables):

- emptot = continuous employer size, measured as number of subscribers and dependents per employer;
- indexmon = calendar index month ("anniversary month") when high-deductible group employers mandated a switch from low-deductible to high-deductible plans; or the corresponding assigned anniversary month for employers that remained in low-deductible plans;
- regc2-regc4 = region of residence (West is the reference, South, Midwest and Northeast);
- age_c4049 = age in years at calendar index month between 40 and 49;
- age_c5059 = age in years at calendar index month between 50 and 59;
- boopcat = category of enrollee baseline out-of-pocket spending (<$500, $500-$999, $1000-$2499, $2500+)
- mem_std_cst1 = indicator for enrollee's total baseline standardized cost being in the first quartile of spending;
- mem_std_cst2 = indicator for enrollee's total baseline standardized cost being in the second quartile of spending;
- mem_std_cst3 = indicator for enrollee's total baseline standardized cost being in the third quartile of spending;
- emp_cost_r1 = enrollee insured through an employer with the lowest category (out of 4 categories) of ratio of total out-of-pocket costs to total standardized costs, an indicator of health plan generosity;
- emp_cost_r2 = enrollee insured through an employer with the second lowest category (out of 4 categories) of ratio of total out-of-pocket costs to total standardized costs, and indicator of health plan generosity;
- emp_cost_r3 = enrollee insured through an employer with the third lowest category (out of 4 categories) of ratio of total out-of-pocket costs to total standardized costs, and indicator of health plan generosity.

(3) b_flag_149 = Indicator for any baseline myocardial infarction;
(4) b_flag_150 = Indicator for any baseline stroke;
(5) b_flag_151 = Indicator for any baseline extremity amputation;
(6) index_yr = Calendar year of the index date of the enrollee's employer;
(7) dm_ind = Indicator for baseline or pre-baseline diabetes diagnosis;
(8) cvd_ind = Indicator for baseline or pre-baseline cardiovascular disease diagnosis;
(9) hyper_ind = Indicator for baseline or pre-baseline hypertension or hyperlipidemia diagnosis;
(10) follow_duration_cat = Categorized number of follow-up months (1-12, 13-24, 25-36, 37+). We included this so that differential dropout on measured characteristics would bias effect estimates.

© 2020 Wharam JF et al. *JAMA Network Open*.

D. Outcome Measures

Our primary outcome was a composite measure at the patient level of the time in months, relative to either the beginning of the baseline period (for baseline measures) or the beginning of the follow-up period (for follow-up measures) until an individual experienced a myocardial infarction or stroke (whichever came first in the relevant period). We constructed our secondary measures of disaggregated myocardial infarction, stroke, and extremity amputation in the same manner. At the patient level, the above measures could occur in the baseline period, the follow-up period (once per person per period), both, or neither given that repeat events can occur. For the composite measure that added death, a given person could only have event during the follow-up period because we required members to be alive during the entire baseline period.

We used validated health insurance claims-based algorithms to define myocardial infarction[11] and stroke.[12] We based our extremity amputation measure on billing procedure codes that are reliable in indicating the occurrence of a reimbursed surgery (eTable 4). We defined myocardial infarction based on detecting a hospitalization of 3-180 days that had International Classification of Diseases, 9th revision (ICD-9-CM) discharge diagnoses in the first or second position as listed in eTable 4.[11] This algorithm has a positive predictive value of 94.1% (95% confidence interval, 93.0%–95.2%). To define stroke, we also used a hospital-based algorithm and ICD-9-CM discharge diagnoses (eTable 4) in the first diagnosis position.[12] This algorithm has a positive predictive value of 92.6% (95% confidence interval, 88.8%-96.4%).

E. Covariates and Subgroups of Interest

We used version 11.1 of the Johns Hopkins ACG® System[13,14] to calculate members' baseline period morbidity score. The algorithm uses age, gender, and ICD-9-CM codes to calculate a morbidity score and the average of the reference population is 1.0.[14] Researchers have validated the index against premature mortality.[13] To derive proxy demographic measures, the data vendor linked members' most recent residential street addresses to their 2010 US Census tract.[15] Census-based measures of socioeconomic status have been validated [16,17] and used in multiple studies to examine the impact of policy changes on disadvantaged populations.[18-20] Using 2008-2012 American Community Survey[21] census tract-level data and validated cut-points,[16,17] we created categories that defined residence in neighborhoods with below-poverty levels of <5%, 5%-9.9%, 10%-19.9%, and ≥20%. Similarly, we defined categories of residence in neighborhoods with below-high-school education levels of <15%, 15%-24.9%, 25%-39.9%, ≥40%.[16,17]

We classified members as from predominantly white, black, or Hispanic neighborhoods if they lived in a census tract with at least 75% of members of the respective race/ethnicity. We then applied a superseding ethnicity assignment using flags created by the E-Tech system (Ethnic Technologies), which analyzes full names and geographic locations of individuals.[22] We classified remaining members as from mixed race/ethnicity neighborhoods. This validated approach of combining surname analysis and census data has positive and negative predictive values of approximately 80 and 90 percent, respectively.[23] Other covariates included age category (40-49 and 50-59, and 50-64 years); sex; US region (West, Midwest, South, Northeast); employer size used as either a continuous variable or with categories of 0-99, 100-999, or >1000 individuals; and calendar month of the index date.

We defined two subgroups of interest for stratified analyses – diabetes and other cardiovascular disease risk factors - using ACG software.[13,14] The ACG algorithm uses ICD-9 and medication billing codes to identify patients with chronic conditions. We first flagged enrollees with diabetes, and among those not in this group, we defined an "other cardiovascular disease risk factor" cohort based on ACG-flagged patients with established cardiovascular disease, hypertension, or hyperlipidemia.

F. Statistical Analyses

We analyzed time to the primary and disaggregated secondary outcomes in Cox proportional hazards regression models adjusted for sex, employer size, race/ethnicity, education, poverty, and US region. The statistical model for estimating adjusted hazard ratios for our measures, at time *t* for an individual, was:

$$\mathrm{h(t,X)} = h_o(t) exp\left(\sum_{k=1}^{p} \beta_k X_k\right)$$

Where:

h(t,X)= hazard function at time t with covariate vector X
$h_o(t)$= The baseline hazard function at time t
$\beta_{0=}$ intercept
$\beta_{k=}$ coefficient for k^{th} characteristic for the patient

© 2020 Wharam JF et al. *JAMA Network Open*.

We implemented this model in Stata version 15.

We analyzed time to event in the baseline and the follow-up periods in a single model and the key term of interest was an interaction between study period (baseline or follow-up) and study group (high-deductible or control). This term generated an adjusted hazard ratio (aHR) of the high-deductible group compared with the control group at follow up versus baseline. An example of the Stata code we used to implement this model and estimate adjusted hazard ratios and produce adjusted marginal survival curves is:

```
stset dxmmonmi [iweight=cem_weights], failure(event_dxmmi)
stcox i.hdhp i.fowup i.hdhp#i.fowup c.emptot i.regc2 i.regc3 i.regc4 i.female i.black i.asian i.hispan i.mixed i.educ2
i.educ3 i.educ4 i.povc2 i.povc3 i.povc4
```

Further explanation of this code is below:

- iweight = cem_weights: incorporates weights from the coarsened exact matching output;
- failure(event_dxmmi) = specifies the modeled outcome, i.e. the dependent variable (myocardial infarction in this case);
- stcox = Stata code that specifies a Cox proportional hazards model;
- i.hdhp = indicates membership in the high-deductible health plan group or control group;
- i.fowup = indicates baseline period or follow-up period;
- i.hdhp#i.fowup = term of interest, interacting the study group and study periods to allow inference about the hazard ratio (HDHP:control) at follow up relative to the hazard ratio (HDHP:control) at baseline;
- c.emptot = continuous employer size, measured as number of subscribers and dependents per employer;
- i.regc2 i.regc3 i.regc4 = U.S. region of residence (West is the reference)
- i.female
- i.black i.asian i.hispan i.mixed = race/ethnicity category (residence in predominantly white neighborhood is the reference)
- i.educ2 i.educ3 i.educ4 = neighborhood education category (highest education level is the reference);
- i.povc2 i.povc3 i.povc4= neighborhood poverty category (lowest poverty level is the reference);

© 2020 Wharam JF et al. *JAMA Network Open*.

II. eResults

eTable 1. Validation of Deductible Imputation Algorithm, Stratified by Employer Size

eTable 1a. Validation of deductible imputation algorithm, using employer accounts of size 75-100 enrollees.

	Gold Standard[a]=high-deductible (n)	Gold Standard=low-deductible (n)
We imputed high-deductible	882,588	24,786
We imputed low-deductible	15,612	511,770

	High-deductible	Low-deductible
Sensitivity	98.3%	95.4%
Specificity	95.4%	98.3%

[a]Gold standard was a benefits variable specific to each employer derived from a benefits table and obtained from the health insurer via the data vendor.

eTable 1b. Validation of deductible imputation algorithm, using employer accounts of size 101-400 enrollees.

	Gold Standard[a]=high-deductible (n)	Gold Standard=low-deductible (n)
We imputed high-deductible	1,998,885	42,655
We imputed low-deductible	20,302	1,748,826

	High-deductible	Low-deductible
Sensitivity	99.0%	97.6%
Specificity	97.6%	99.0%

[a]Gold standard was a benefits variable specific to each employer derived from a benefits table and obtained from the health insurer via the data vendor.

© 2020 Wharam JF et al. *JAMA Network Open*.

eTable 1c. Validation of deductible imputation algorithm, using employer accounts of size 401-700 enrollees.

	Gold Standard[a]=high-deductible (n)	Gold Standard=low-deductible (n)
We imputed high-deductible	83,393	485
We imputed low-deductible	2,017	122,983

	High-deductible	Low-deductible
Sensitivity	97.6%	99.6%
Specificity	99.6%	97.6%

[a]Gold standard was a benefits variable specific to each employer derived from a benefits table and obtained from the health insurer via the data vendor.

eTable 1d. Validation of deductible imputation algorithm, using employer accounts of size 701-1000 enrollees.

	Gold Standard[a]=high-deductible (n)	Gold Standard=low-deductible (n)
We imputed high-deductible	9950	0
We imputed low-deductible	0	19,664

	High-deductible	Low-deductible
Sensitivity	100.0%	100.0%
Specificity	100.0%	100.0%

[a]Gold standard was a benefits variable specific to each employer derived from a benefits table and obtained from the health insurer via the data vendor.

© 2020 Wharam JF et al. *JAMA Network Open*.

eFigure 1. Study Design Showing Example Members of the High-Deductible Health Plan Group and Matched Control Group

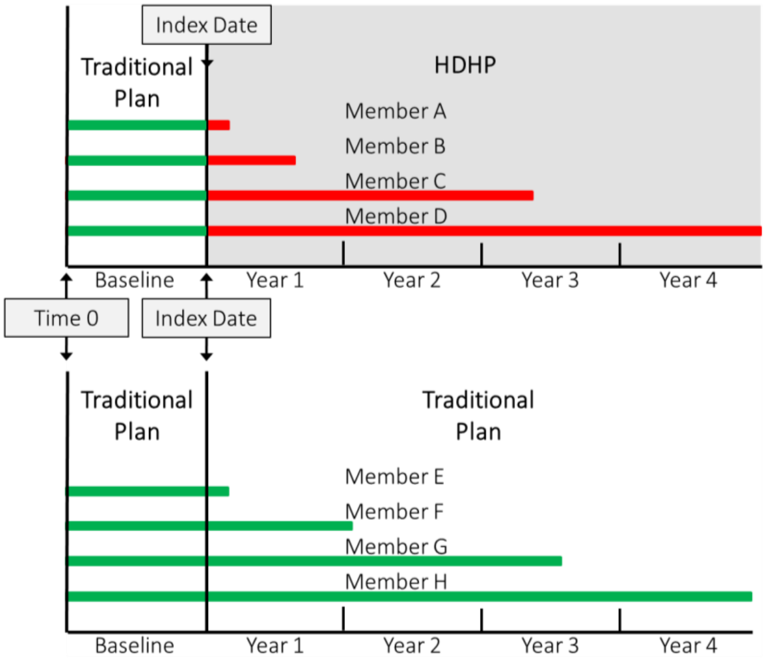

© 2020 Wharam JF et al. *JAMA Network Open*.

eTable 2. Baseline Characteristics of the Subgroup with Diabetes[1] before and after Matching

	Unmatched					Matched				
	**HDHP Group**		**Control Group**		**SDiff[2]**	**HDHP Group**		**Control Group**		**SDiff[1]**
	**(N=30,730)**		**(N=335,325)**			**(N=30,449)**		**(N=292,170)**		
**ACG score, Mean (SD)[2]**	1.9	(2.9)	2.0	(3.0)	-0.028	1.9	(2.9)	1.9	(2.7)	0.014
**Age, Mean yrs (SD)**	53.4	(6.7)	53.8	(7.0)	-0.055	53.4	(6.7)	53.6	(6.9)	-0.028
**Outpatient copayment, Median $ (SD)**	19.3	(6.2)	18.6	(6.4)	0.388	19.3	(6.2)	18.6	(6.4)	0.081
**Female, No. (%)**	13,533	(44.0)	154,212	(46.0)	-0.039	13,421	(44.1)	128,894	(44.1)	-0.001
**No. (%) living in neighborhoods with below- poverty levels[3] of**										
<5.0%	6,028	(19.6)	71,878	(21.4)	0.046	5,967	(19.6)	58,836	(20.1)	0.026
5.0%-9.9%	7,680	(25.0)	85,891	(25.6)		7,625	(25.0)	75,503	(25.8)	
10.0%-19.9%	9,993	(32.5)	104,860	(31.3)		9,927	(32.6)	94,499	(32.3)	
≥20%	6,959	(22.6)	72,309	(21.6)		6,925	(22.7)	63,312	(21.7)	
Missing Poverty	70	(0.2)	387	(0.1)		5	(0.0)	20	(0.0)	
**No. (%) living in neighborhoods with below-high-school education levels[3] of**										
<15%	19,753	(64.3)	221,205	(66.0)	0.049	19,598	(64.4)	193,534	(66.2)	0.071
15%-24.9%	7,000	(22.8)	71,988	(21.5)		6,959	(22.9)	63,202	(21.6)	
25%-39.9%	3,054	(9.9)	33,331	(9.9)		3,039	(10.0)	28,395	(9.7)	
>=40%	854	(2.8)	8,420	(2.5)		849	(2.8)	7,022	(2.4)	
Missing Education	69	(0.2)	381	(0.1)		4	(0.0)	17	(0.0)	
**Race/ethnicity, No. (%)[4]**										
Asian	855	(2.8)	11,320	(3.4)	0.119	840	(2.8)	8,110	(2.8)	0.038
Black Neighborhood	957	(3.1)	14,439	(4.3)		955	(3.1)	9,197	(3.1)	
Hispanic	3,385	(11.0)	36,965	(11.0)		3,336	(11.0)	30,469	(10.4)	
Mixed Neighborhood	6,573	(21.4)	82,634	(24.6)		6,534	(21.5)	64,787	(22.2)	
White Neighborhood	18,913	(61.5)	189,686	(56.6)		18,783	(61.7)	179,591	(61.5)	
Missing race/ethnicity	47	(0.2)	281	(0.1)		1	(0.0)	16	(0.0)	
**Age Categories, No. (%)**										
40-49	9,146	(29.8)	96,736	(28.8)	0.070	9,075	(29.8)	86,375	(29.6)	0.025
50-59	14,543	(47.3)	150,797	(45.0)		14,414	(47.3)	134,330	(46.0)	
50-64	7,041	(22.9)	87,792	(26.2)		6,696	(22.9)	71,465	(24.5)	
**ACG Categories,[5] No.**										
ACG Score 0-1	113,507	(44.0)	140,420	(41.9)	0.055	13,357	(43.9)	126,425	(42.3)	0.023
ACG Score 1.01-2	10,066	(32.8)	112,010	(33.4)		10,027	(32.9)	98,266	(33.6)	
ACG Score 2.01-3	1,446	(4.7)	17,347	(5.2)		1,439	(4.7)	14,275	(4.9)	
ACG Score 3.01-4	3,097	(10.1)	35,586	(10.6)		3,064	(10.1)	29,696	(10.2)	
ACG Score 4.01+	2,614	(8.5)	29,962	(8.9)		2,562	(8.4)	23,508	(8.0)	
**United States region,**										
South	15,188	(49.4)	159,978	(47.7)	0.0193	15,068	(49.5)	153,896	(52.7)	0.105
West	2,859	(9.3)	40,383	(12.0)		2,841	(9.3)	27,032	(9.3)	
Midwest	10,590	(34.5)	98,978	(29.4)		10,528	(34.6)	95,524	(32.7)	
Northeast	2,028	(6.6)	35,645	(10.6)		2,012	(6.6)	15,719	(5.4)	
Missing	65	(0.0)	341	(0.1)						
**Out-of-Pocket Categories, No. (%)**										
$0.00-$500.00	7,208	(23.5)	92,352	(27.5)	0.197	7,114	(23.4)	67,155	(23.0)	0.055
$500.01-$999.99	7,156	(23.3)	88,598	(26.4)		7,111	(23.4)	70,286	(24.1)	
$1,000.00-$2,499.99	10,918	(35.5)	112,845	(33.7)		10,848	(35.6))	107,637	(36.8)	
≥$2,500.00	5,448	(17.7)	41,530	(12.4)		5,376	(17.7)	47,091	(16.1)	
**Employer size, No. enrollees (%)**										
0-99	19,079	(61.2)	61,714	(18.4)	1.310	18,868	(62.0)	177,688	(60.8)	0.085
100-999	10,196	(33.2)	111,933	(33.4)		10,126	(33.3)	92,758	(31.7)	
1000+	1,455	(4.7)	161,678	(48.2)		1,455	(4.8)	21,724	(7.4)	

Abbreviations: HDHP, high-deductible health plan; SDiff, standardized difference; SD, standard deviation. [1] Based on Johns Hopkins ACG software definition of diabetes. [2]Standardized difference closer to 0 indicates greater similarity. [3]Based on 2008-2012 American Community Survey at the Census Tract level. [4]Race/ethnicity definitions available in manuscript. [5]Based on Johns Hopkins ACG Software; the mean score in the overall sample (members in and not in this cohort) was 0.62 to 0.82 from 2003 – 2014.

© 2020 Wharam JF et al. *JAMA Network Open*.

eTable 3. Baseline Characteristics of the Subgroup with Established Cardiovascular Disease, Hypertension, or Hyperlipidemia[1] before and after Matching

	Unmatched					Matched				
	HDHP Group		Control Group		SDiff[2]	HDHP Group		Control Group		SDiff[1]
	(N=128,627)		(N=1,349,780)			(N=127,667)		(N=1,203,581)		
**ACG score, Mean (SD)[2]**	1.4	(2.1)	1.4	(2.1)	-0.012	1.4	(2.1)	1.4	(2.0)	0.002
**Age, Mean yrs (SD)**	52.5	(6.7)	52.7	(6.9)	-0.018	52.5	(6.7)	52.7	(6.8)	-0.028
**Outpatient copayment, Median $ (SD)**	19.3	(6.2)	18.6	(6.4)	0.383	19.3	(6.2)	18.6	(6.4)	0.101
**Female, No. (%)**	60,767	(47.2)	657,532	(48.7)	-0.029	60,322	(47.2)	569,653	(47.3)	-0.002
**No. (%) living in neighborhoods with below-poverty levels[3] of**										
<5.0%[4]	32,409	(25.2)	368,100	(27.3)	0.050	32,190	(25.2)	308,038	(25.6)	0.032
5.0%-9.9%[4]	35,530	(27.6)	377,857	(28.0)		35,288	(27.6)	338,508	(28.1)	
10.0%-19.9%[5]	38,635	(30.0)	388,591	(28.8)		38,418	(30.1)	362,869	(30.1)	
≥20%[5]	21,860	(17.0)	213,884	(15.8)		21,762	(17.0)	193,982	(16.1)	
Missing Poverty	193	(0.2)	1,348	(0.1)		9	(0.0)	182	(0.0)	
**No. (%) living in neighborhoods with below-high-school education levels[3] of**										
<15%	93,757	(72.9)	998,402	(74.0)	0.083	93,163	(73.0)	891,972	(74.1)	0.083
15%-24.9%	23,461	(18.2)	236,441	(17.5)		23,326	(18.3)	213,337	(17.7)	
25%-39.9%	9,238	(7.2)	94,688	(7.0)		9,200	(7.2)	81,880	(6.8)	
>=40%	1,981	(1.5)	18,926	(1.4)		1,972	(1.5)	16,231	(1.3)	
Missing Education	190	(0.1)	1,323	(0.1)		6	(0.0)	159	(0.0)	
**Race/ethnicity, No. (%)[4]**										
Asian	2,683	(2.1)	37,189	(2.8)	0.139	2,658	(2.1)	25,364	(2.1)	0.026
Black Neighborhood	2,585	(2.0)	38,235	(2.8)		2,572	(2.0)	25,064	(2.1)	
Hispanic	8,740	(6.8)	101,919	(7.6)		8,645	(6.8)	81,810	(6.8)	
Mixed Neighborhood	24,937	(19.4)	297,844	(22.1)		24,803	(19.4)	240,006	(19.9)	
White Neighborhood	89,532	(69.6)	873,530	(64.7)		88,983	(69.7)	831,193	(69.1)	
Missing race/ethnicity	150	(0.1)	1,063	(0.1)		6	(0.0)	143	(0.0)	
**Age Categories, No. (%)**										
40-49	44,415	(34.5)	470,914	(34.9)	0.055	44,174	(34.6)	410,233	(34.1)	0.051
50-59	60,588	(47.1)	602,138	(44.6)		60,127	(47.1)	554,463	(46.1)	
50-64	23,624	(18.4)	276,728	(20.5)		23,366	(18.3)	238,885	(19.8)	
**ACG Categories, No. (%)[5]**										
ACG Score 0-1	67,186	(52.2)	686,165	(50.8)	0.119	66,511	(52.1)	619,254	(51.5)	0.039
ACG Score 1.01-2	40,795	(31.7)	439,363	(32.6)		40,646	(31.8)	388,806	(32.3)	
ACG Score 2.01-3	5,518	(4.3)	61,663	(4.6)		5,484	(4.3)	53,466	(4.4)	
ACG Score 3.01-4	9,332	(7.3)	102,018	(7.6)		9,282	(7.3)	90,388	(7.5)	
ACG Score 4.01+	5,796	(4.5)	60,571	(4.5)		5,744	(4.5)	51,666	(4.3)	
**United States region, No. (%)**										
South	62,364	(48.5)	610,119	(45.2)	0.245	61,987	(48.6)	620,792	(51.6)	0.067
West	12,306	(9.6)	171,017	(12.7)		12,204	(9.6)	122,518	(10.2)	
Midwest	44,619	(34.7)	402,086	(29.8)		44,386	(34.8)	389,860	(32.4)	
Northeast	9,154	(7.1)	165,430	(12.3)		9,090	(7.1)	70,411	(5.9)	
Missing	184	(0.1)	1,128	(0.1)						
**Out-of-Pocket Categories, No. (%)**										
$0.00-$500.00	50,736	(39.4)	611,852	(45.3)	0.209	50,255	(39.4)	472,376	(39.2)	0.069
$500.01-$999.99	31,277	(24.3)	345,699	(25.6)		31,129	(24.4)	305,753	(25.4)	
$1,000.00-$2,499.99	33,903	(26.4)	304,379	(22.6)		33,694	(26.4)	319,168	(26.5)	
≥$2,500.00	12,711	(9.9)	87,850	(6.5)		12,589	(9.9)	106,284	(8.8)	
**Employer size, No. enrollees (%)**										
0-99	83,665	(65.0)	263,853	(19.5)	1.312	82,884	(64.9)	762,238	(63.3)	0.170
100-999	39,290	(30.5)	440,854	(32.7)		39,116	(30.6)	347,356	(28.9)	
1000+	5,672	(4.4)	645,073	(47.8)		5,667	(4.4)	93,987	(7.8)	

Abbreviations: HDHP, high-deductible health plan; SDiff, standardized difference; SD, standard deviation. [1] Based on Johns Hopkins ACG software definitions of cardiovascular disease, hypertension, or hyperlipidemia. [2]Standardized difference closer to 0 indicates greater similarity. [3]Based on 2008-2012 American Community Survey at the Census Tract level. [4]Race/ethnicity definitions available in manuscript. [5]Based on Johns Hopkins ACG Software; the mean score in the overall sample (members in and not in this cohort) was 0.62 to 0.82 from 2003 – 2014.

© 2020 Wharam JF et al. *JAMA Network Open*.

eTable 4. Billing Codes Used to Create Measures of Myocardial Infarction, Stroke, and Extremity Amputation

Category	ICD-9-CM Diagnosis or Procedure Codes and CPT-4 Codes
Myocardial Infarction	ICD-9 codes: 410.01, 410.11, 410.21, 410.31, 410.41, 410.51, 410.61, 410.71, 410.81, or 410.91
Stroke	ICD-9 codes: 430, 431, 433.01, 433.11, 433.21, 433.31, 433.41, 433.51, 433.61, 433.71, 433.81, 433.91, 434.01, 434.11, 434.21, 434.31, 434.41, 434.51, 434.61, 434.71, 434.81, 434.91 or 436
Extremity Amputation	CPT codes: 01404, 23900, 23920, 24900, 24920, 24931, 25900, 25905, 25915, 25920, 25922, 25927, 26910, 26951, 26952, 27290, 27295, 27590, 27591, 27592, 27598, 27880, 27881, 27882, 27888, 27889, 28800, 28805, 28810, 28820, 28825; ICD-9 procedure codes: 840, 841, 840.0, 840.1, 840.2, 840.3, 841.0, 841.1, 841.2, 841.3, 841.4, 841.5, 841.6, 841.7, 841.8, 841.9

Abbreviations: CPT, Current Procedural Terminology; ICD-9-CM, International Classification of Diseases, 9th edition, clinical modification

© 2020 Wharam JF et al. *JAMA Network Open*.

eTable 5. Out-of-Pocket Total (Medical and Pharmacy), Medical, and Pharmacy Expenditure Changes in the HDHP and Control Groups in Follow-up Years 1-4 Compared with the Baseline Year

	**Mean Out-of-pocket Expenditure, $[1]**										**Relative Change vs Baseline, HDHP versus Control Group, % (95% Confidence Interval)**											
	**HDHP Group**					**Control Group**																
	**Baseline**	**Year 1**	**Year 2**	**Year 3**	**Year 4**	**Baseline**	**Year 1**	**Year 2**	**Year 3**	**Year 4**	**Year 1**			**Year 2**			**Year 3**			**Year 4**		
Total	1160.0	1554.5	1619.7	1706.9	1837.8	1098.7	1171.7	1210.8	1269.3	1327.4	**25.7**	**(24.2,**	**27.1)**	**26.7**	**(25.2,**	**28.2)**	**27.4**	**(25.5,**	**29.3)**	**31.1**	**(28.3,**	**33.9)**
Medical	637.8	958.5	992.8	1059.4	1170.8	584.9	623.0	633.2	664.3	705.5	**41.1**	**(38.3,**	**43.9)**	**43.8**	**(41.0,**	**46.5)**	**46.3**	**(42.9,**	**49.7)**	**52.2**	**(47.2,**	**57.2)**
Pharmacy	522.2	595.1	625.5	647.7	668.6	513.8	548.8	577.2	605.2	622.5	**6.7**	**(6.3,**	**7.1)**	**6.6**	**(5.8,**	**7.4)**	**5.3**	**(4.1,**	**6.5)**	**5.7**	**(4.0,**	**7.4)**

Abbreviations: HDHP, high-deductible health plan. [1]Values derives using generalized estimating equations with a zero-inflated negative binomial distribution and adjusted for sex, employer size, race/ethnicity, education, poverty, and US region.

© 2020 Wharam JF et al. *JAMA Network Open*.

eTable 6. Cardioprotective Medication Fills per Year in the High-Deductible Health Plan and Control Groups in Follow-up Years 1-4 Compared with the Baseline Year

Medication Class	Mean 30-Day Fills										Relative Change vs Baseline, HDHP versus Control Group, % (95% Confidence Interval)[1]							
	HDHP Group					Control Group					Year 1		Year 2		Year 3		Year 4	
	Baseline	Year 1	Year 2	Year 3	Year 4	Baseline	Year 1	Year 2	Year 3	Year 4								
Antihypertensives[2]	7.3	7.7	8.0	8.2	8.5	7.4	8.0	8.4	8.9	9.1	**-2.0**	**(-2.4, -1.6)**	**-3.5**	**(-4.3, -2.7)**	**-5.4**	**(-6.6, -4.2)**	**-5.7**	**(-7.3, -4.1)**
Lipid Lowering Agents[2]	3.5	3.8	4.1	4.3	4.6	3.6	4.0	4.4	4.7	4.9	**-2.8**	**(-3.3, -2.2)**	**-3.7**	**(-4.6, -2.7)**	**-5.0**	**(-6.4, -3.6)**	**-5.1**	**(-7.0, -3.2)**
Oral Antidiabetics[3]	1.5	1.6	1.7	1.8	1.8	1.5	1.7	1.8	1.9	2.0	**-2.2**	**(-3.1, -1.2)**	**-3.0**	**(-5.0, -1.0)**	**-3.9**	**(-6.9, -0.9)**	**-4.6**	**(-8.7, -0.6)**
Insulin[3]	0.3	0.3	0.3	0.4	0.4	0.3	0.3	0.4	0.4	0.4	**-2.1**	**(-4.1, -0.1)**	-3.0	(-7.0, 1.0)	-1.5	(-7.4, 4.4)	-3.6	(-11.4, 4.3)

Abbreviations: HDHP, high-deductible health plan. [1]Values derives using generalized estimating equations with a zero-inflated negative binomial distribution and adjusted for sex, employer size, race/ethnicity, education, poverty, and US region. [2]Among entire cohort. [3]Among diabetes cohort.

© 2020 Wharam JF et al. *JAMA Network Open*.

eTable 7. Adjusted Hazard Ratios for Sensitivity Analyses Examining a Very High-Risk Subgroup and a Cohort with Index Dates in 2009 and After

Subgroup	Hazard Ratio	(95% Confidence Interval)
Very high-risk subgroup (diabetes, cardiovascular disease, and either hypertension or hyperlipidemia)		
Myocardial infarction or Stroke	0.76	(0.57, 1.03)
Myocardial infarction	0.77	(0.55, 1.08)
Stroke	0.77	(0.35, 1.69)
Amputation	1.09	(0.60, 1.97)
Cohort with index dates in 2009 and after		
Myocardial infarction or Stroke	1.07	(0.87, 1.32)
Myocardial infarction	1.08	(0.83, 1.41)
Stroke	1.07	(0.76, 1.50)
Amputation	1.00	(0.62, 1.61)

See manuscript for details of subgroup construction, measure details, and statistical models

© 2020 Wharam JF et al. *JAMA Network Open*.

eTable 8. Adjusted Hazard Ratios for Sensitivity Analyses that Exclude HDHP Members with Health Savings Accounts or Health Reimbursement Arrangements

	**Hazard Ratio**	**(95% Confidence Interval)**	
Overall Cohort, excluding members in Health Savings Account or Health Reimbursement Arrangement plans			
Myocardial infarction or Stroke	1.04	(0.92,	1.18)
Myocardial infarction	1.06	(0.91,	1.24)
Stroke	1.02	(0.81,	1.28)
Amputation	0.97	(0.71,	1.32)
Cohort with Diabetes, excluding members in Health Savings Account or Health Reimbursement Arrangement plans			
Myocardial infarction or Stroke	0.97	(0.77,	1.22)
Myocardial infarction	0.99	(0.75,	1.30)
Stroke	0.95	(0.63,	1.42)
Amputation	1.01	(0.69,	1.49)
Cohort with Other Cardiovascular Risk Factors, excluding members in Health Savings Account or Health Reimbursement Arrangement plans			
Myocardial infarction or Stroke	0.98	(0.85,	1.14)
Myocardial infarction	1.02	(0.85,	1.22)
Stroke	0.96	(0.73,	1.26)
Amputation	1.10	(0.69,	1.76)

See manuscript for details of subgroup construction, measure details, and statistical models

© 2020 Wharam JF et al. *JAMA Network Open*.

eFigure2. Weighted and Adjusted Cumulative Rates of First Major Cardiovascular Events[a] and All-Cause Mortality in the HDHP and Control Groups

(a) Overall Cohort[b]

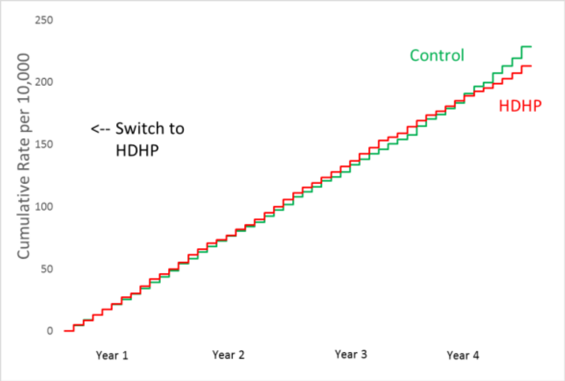

Adjusted hazard ratio, follow-up vs baseline: 0.98 (0.93, 1.04)

(b) Diabetes Cohort[c]

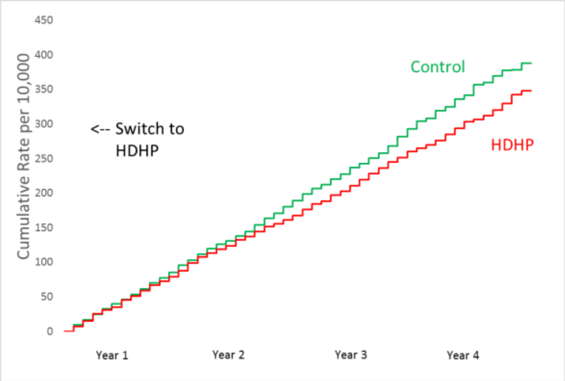

Adjusted hazard ratio, follow-up vs baseline: 0.91 (0.83, 1.01)

(c) Other Cardiovascular Risk Factor Cohort[d]

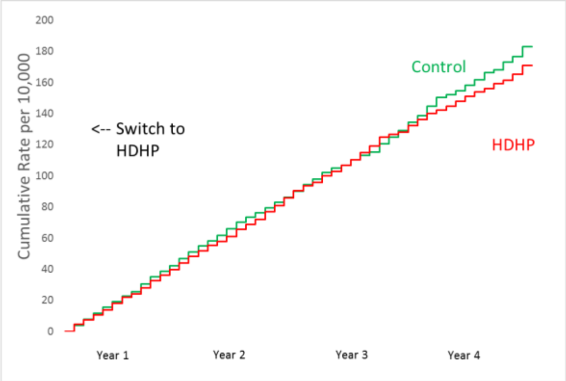

Adjusted hazard ratio, follow-up vs baseline: 0.95 (0.89, 1.02)

Abbreviation: HDHP, high-deductible health plan. [a]Comprising myocardial infarction or stroke [b]Includes patients with diabetes, cardiovascular disease, hypertension, or hyperlipidemia diagnosed before the index date. [c]Defined using ACG software and diagnosed before the index date. [d]Comprising patients with cardiovascular disease, hypertension, or hyperlipidemia based on ACG software and diagnosed before the index date. **Note**: Plots include only time after the index date (i.e., follow-up time only) given that the outcome measure includes death and we required members to be alive for the entire baseline period.

© 2020 Wharam JF et al. *JAMA Network Open*.

eReferences

1. The Kaiser Family Foundation Employer Health Benefits 2018 Annual Survey. 2018 [Available from: http://files.kff.org/attachment/Report-Employer-Health-Benefits-Annual-Survey-2018.
2. Hallberg K, Cook TD, Steiner PM, Clark MH. Pretest Measures of the Study Outcome and the Elimination of Selection Bias: Evidence from Three Within Study Comparisons. Prev Sci. 2018;19(3):274-83.
3. St.Clair T, Cook TD, Hallberg K. Examining the Internal Validity and Statistical Precision of the Comparative Interrupted Time Series Design by Comparison With a Randomized Experiment. American Journal of Evaluation. 2014;35(3):311-27.
4. Iacus S, King G, Porro G. CEM: Coarsened Exact Matching Software [Available from: https://gking.harvard.edu/cem.
5. Iacus SM, King G, Porro G. Causal Inference Without Balance Checking: Coarsened Exact Matching. Political Analysis. 2012;20(1):1--24.
6. Iacus SM, King G, Porro G. Multivariate Matching Methods That Are Monotonic Imbalance Bounding. Journal of the American Statistical Association. 2011;106(493):345-61.
7. Schreyogg J, Stargardt T, Tiemann O. Costs and quality of hospitals in different health care systems: a multi-level approach with propensity score matching. Health Econ. 2011;20(1):85-100.
8. Wharam JF, Zhang F, Landon BE, LeCates R, Soumerai S, Ross-Degnan D. Colorectal Cancer Screening in a Nationwide High-deductible Health Plan Before and After the Affordable Care Act. Medical care. 2016;54(5):466-73.
9. Iacus SM KG, Porro G. . Multivariate matching methods that are monotonic imbalance bounding. J Am Stat Assoc. 2011;106(493):345-61.
10. Blackwell M, Iacus S, King G, Porro G. cem: Coarsened exact matching in Stata. Stata Journal. 2009;9(4):524-46.
11. Kiyota Y, Schneeweiss S, Glynn RJ, Cannuscio CC, Avorn J, Solomon DH. Accuracy of Medicare claims-based diagnosis of acute myocardial infarction: estimating positive predictive value on the basis of review of hospital records. Am Heart J. 2004;148(1):99-104.
12. Kumamaru H, Judd SE, Curtis JR, Ramachandran R, Hardy NC, Rhodes JD, et al. Validity of claims-based stroke algorithms in contemporary Medicare data: reasons for geographic and racial differences in stroke (REGARDS) study linked with medicare claims. Circ Cardiovasc Qual Outcomes. 2014;7(4):611-9.
13. Reid RJ, Roos NP, MacWilliam L, Frohlich N, Black C. Assessing population health care need using a claims-based ACG morbidity measure: a validation analysis in the Province of Manitoba. Health services research. 2002;37(5):1345-64.
14. ACG. The Johns Hopkins ACG System [Available from: https://www.hopkinsacg.org/advantage/.
15. USCensus. Census 2000 Gateway: U.S. Bureau of the Census; 2000 [Available from: https://www.census.gov/main/www/cen2000.html.
16. Krieger N. Overcoming the absence of socioeconomic data in medical records: validation and application of a census-based methodology. American Journal of Public Health. 1992;82(5):703-10.
17. Krieger N, Chen JT, Waterman PD, Rehkopf DH, Subramanian SV. Race/ethnicity, gender, and monitoring socioeconomic gradients in health: a comparison of area-based socioeconomic measures--the public health disparities geocoding project. American Journal of Public Health. 2003;93(10):1655-71.
18. Trivedi AN, Zaslavsky AM, Schneider EC, Ayanian JZ. Relationship between quality of care and racial disparities in Medicare health plans. Jama. 2006;296(16):1998-2004.
19. Trivedi AN, Rakowski W, Ayanian JZ. Effect of cost sharing on screening mammography in medicare health plans. The New England journal of medicine. 2008;358(4):375-83.
20. Selby JV, Fireman BH, Swain BE. Effect of a copayment on use of the emergency department in a health maintenance organization. New England Journal of Medicine. 1996;334(10):635-41.
21. American Community Survey [Available from: https://www.census.gov/programs-surveys/acs/.
22. Ethnic Technologies. Frequently Asked Questions [Available from: www.ethnictechnologies.com/faq.
23. Fiscella K, Fremont AM. Use of geocoding and surname analysis to estimate race and ethnicity. Health services research. 2006;41(4 Pt 1):1482-500.

© 2020 Wharam JF et al. *JAMA Network Open*.
